# Structure, form, and meaning in the mental lexicon: evidence from Arabic

**DOI:** 10.1080/23273798.2015.1048258

**Published:** 2015-09-28

**Authors:** Sami Boudelaa, William D. Marslen-Wilson

**Affiliations:** ^a^ Department of Linguistics, United Arab Emirates University, Al Ain, UAE; ^b^ Department of Experimental Psychology, University of Cambridge, Cambridge, UK; ^c^ MRC Cognition and Brain Sciences Unit, Cambridge, UK

**Keywords:** morphological processing, mental lexicon, Arabic, roots, word patterns, semantic and phonological effects

## Abstract

Does the organization of the mental lexicon reflect the combination of abstract underlying morphemic units or the concatenation of word-level phonological units? We address these fundamental issues in Arabic, a Semitic language where every surface form is potentially analyzable into abstract morphemic units – the word pattern and the root – and where this view contrasts with stem-based approaches, chiefly driven by linguistic considerations, in which neither roots nor word patterns play independent roles in word formation and lexical representation. Five cross-modal priming experiments examine the processing of morphologically complex forms in the three major subdivisions of the Arabic lexicon – deverbal nouns, verbs, and primitive nouns. The results demonstrate that root and word pattern morphemes function as abstract cognitive entities, operating independently of semantic factors and dissociable from possible phonological confounds, while stem-based approaches consistently fail to accommodate the basic psycholinguistic properties of the Arabic mental lexicon.

The representation of words in the mind is an unresolved challenge for theories of human language function. There persist fundamental questions about the basic properties of these representations – are they structured in terms of words or morphemes, do they have abstract linguistic properties that go beyond the interaction of form-based and semantic constraints, and how far do they obey similar organisational principles across different languages? Over the years, almost every conceivable answer to these questions has appeared in the psycholinguistic literature (e.g., Baayen et al., 2011; Butterworth, 1983; Caramazza, Laudanna, & Romani, 1988; Marslen-Wilson, Tyler, Waksler, & Older, 1994; Plaut & Gonnerman, 2000; Rastle & Davis, 2008; Schreuder & Baayen, 1995; Taft & Forster, 1975), accompanied by an equally vast and diverse volume of empirical research.

Despite the important achievements of this body of research and theory, one limitation is that it is primarily informed by studies run on English and other Indo-European languages, and lacks the typological range necessary to capture the universal characteristics of human language processing. To address this we need to investigate issues of morphological structure and function in non-Indo-European contexts, in languages with qualitatively different mechanisms of word formation. Complementing earlier behavioural studies in non-Indo-European languages including Finnish (e.g., Järvikivi, Pyykkönen, & Niemi, 2009), Chinese (e.g., Zhou & Marslen-Wilson, 1994), and Hebrew (e.g., Frost, Forster, & Deutsch, 1997), here we focus on a Semitic language, Arabic, that is standardly thought to be based on non-concatenative word building procedures, contrasting sharply with the concatenative (stem + affix) procedures that dominate word formation in Indo-European languages. Arabic consonantal roots, conveying semantic information, are interleaved with word patterns that express phonological and morpho-syntactic information. This historically preferred root and pattern analysis of Arabic has, nonetheless, been substantially challenged by an analysis of Arabic morphology (and of Semitic morphology more generally) as a stem-based system that uses the concatenative affixation processes that are dominant cross-linguistically (e.g., Benmamoun, 1999, 2003; Hammond, 1988; Heath, 1987, 2003; McCarthy & Prince, 1990; Ratcliffe, 1998, 2004).

Here we consider the viability of these two approaches to Arabic in terms of their applicability to psycholinguistic studies of the Arabic mental lexicon. To do so, we use the cross-modal priming paradigm to ask how central lexical representations, the abstract targets of both visual and auditory input, are structurally organised. Priming effects between a pair of words depends on the presence of an underlying representational link between prime and target. We ask whether a representational linkage in terms of shared word patterns or roots is sufficient to generate priming, or whether successful priming requires the presence of a shared stem.

We begin with an overview of the relevant aspects of Arabic morphology followed by a review of existing research on the processing and representation of Semitic word patterns and roots. We then report five cross-modal priming experiments that cover deverbal nouns, verbs, and primitive nouns, constituting the major categories of the Arabic lexical system. This provides a uniquely broad coverage of a Semitic lexical system viewed in psycholinguistic terms, while allowing the relative merits of root-pattern and stem-based accounts to be assessed in this rich cognitive processing context.

## Arabic morphology

The historically predominant analysis of Arabic morphology is stated in root-and-pattern terms, where surface word forms are constructed by interleaving bound morphemes one within the other. We will use this approach here to present an initial overview of Arabic morphology.

Within the root and pattern framework, almost every phonetic form in the language is thought to consist of at least two morphemes, a *root* and a *word pattern* (Cantineau, 1950; Cohen, 1951, 1961; Hilaal, 1990; Holes, 1995). The root usually consists of three consonants while the word pattern is made up of vowels but can contain consonants as well (Hilaal, 1990; Wright, 1995). The root consonants carry a semantic core meaning shared to various degrees by most of the derivatives of a given root while the word pattern provides information about the phonological structure of the surface form and its morpho-syntactic properties. For example the surface form [xara
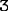
a] *go out* consists of the root morpheme {xr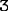} with the general semantic load *going out* and the word pattern {fa
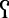
ala}^1^ with the syntactic reading *singular, active*.

The process of combining consonantal roots with word patterns to yield surface forms is very productive. While languages with a concatenative morphology, such as English or French, will use different morphemes to encode notions belonging to the same semantic field, Arabic employs the same root consonants combined with different word patterns. Consider the Arabic words [kataba], [kaataba], [maktabun], [maktabatun], [kitaabun], [maktuubun], and [kuttaabun]. These all have in common the root {ktb}, with the semantic field of writing, which makes them morphologically, phonologically and to various degrees semantically related. Their respective English counterparts – *write*, *correspond*, *office*, *library*, *book*, *destiny,* and *Koran school* – may be semantically or associatively related, but share neither morphological nor phonological relationships.

Word pattern morphemes are also highly productive. For instance, the syntactic reading *agentive* of the verbal word pattern {faa
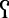
ilun} will be realised transparently in any combination of this pattern with a transitive root – for example with {ktb} to obtain [kaatibun] *one who writes*, or with {ktm} to yield [kaatimun] *one who conceals* and so forth. Word pattern morphemes can have distinct phonological structures but common syntactic properties. The word patterns {faa
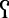
ilun} and {mufta
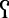
ilun} for example, both carry the syntactic reading *agentive*, leading to forms like [kaatimun] *one who conceals* and [mumtaħinun] *one who examines*. Conversely, the same word pattern can have different readings depending on the type of root it combines with. The pattern {fi
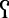
aalatun}, for example, typically denotes a *singular profession* noun in forms like [ħilaaqatun] *hair dressing*, [ti
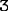
aaratun] *trade*, and [kitaabatun] *writing* (i.e., profession of being a writer). However, the same word pattern has only a feminine derived noun reading in a number of forms such as [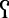
ibaaratun] *expression*, [ħikaayatun] *story,* and [binaayatun] *building*, where there is no trace of the “profession noun” meaning.

The orthographic system of Arabic does not treat roots and word patterns on an equal footing. Arabic script is primarily consonantal and represents consonants and long vowels by letters, while short vowels are marked by diacritics (similar to the system of “pointing” in Hebrew script). In normal texts intended for adult readers, the diacritics are omitted. This means that roots are always fully realised as part of the orthographic string, but that the vocalic components of word patterns are not, unless they involve one of the three long vowels /aa, uu, ii/. For instance, because there is no letter for the sound /a/, the word for *hide* (pronounced [katama]) is typically written as [ktm]. Written Arabic can therefore involve a significant amount of ambiguity and the identity of word patterns will often have to be inferred based on the consonants of the root (Boudelaa, 2014; Boudelaa & Marslen-Wilson, 2005).

Although these characteristics of roots and word patterns are claimed to hold true of Arabic surface word forms in general, there are significant differences in the way they apply to the different components of Arabic morphology. This makes a basic distinction between primitive noun morphology and verb morphology, where the latter includes both verbs proper and nouns related to verbs, known as deverbal nouns (Bohas & Guillaume, 1984; Holes, 1995; Wright, 1995). We provide below a brief description of these three different components of the Arabic morphological system, highlighting the questions they raise for potential models of morphological processing and representation.

### Deverbal nouns

There are around 500 deverbal nominal word patterns in Arabic (Boudelaa & Marslen-Wilson, 2010). When a surface form is a deverbal noun, it is usually straightforward to factor out the respective contributions of the root and the word pattern. The meaning of the deverbal noun [maktabun], glossable in English as *place for writing* (or *office*), derives from the combination of the meaning of the root {ktb} *writing* with the morpho-syntactic value of the word pattern {maf
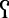
alun} *place noun*, *singular*, *masculine*. Similarly, the meaning of the surface form [miknasatun] *broom* is straightforwardly linked to the meaning of the root {kns} *cleaning* and to the properties of the word pattern {mif
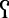
alatun} indicating *instrument noun, singular, feminine*.

However, the interpretation of a particular nominal word pattern is not transparent across the board. Most word patterns are associated with two or more morpho-syntactic functions, disambiguated in the context of the specific root that the pattern combines with. For instance, the word pattern {fa
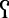
iilun} has the potential readings *intensive noun* (i.e., a noun that expresses intensity of action or repeated action) and *a common noun* (i.e., a noun derived from a triliteral root where the word pattern does not carry specific additional functions such as *place noun* or *profession noun* (see Ryding, 2005). This pattern will surface with the common noun role when combined with a polysemous verbal root like {qs^
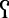
^d} *intention/poetry*, or {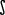
rt^
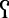
^} *condition/ribbon*, to yield the surface forms [qas^
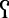
^iidun] *poem*, and [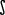
ariit^
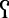
^un] *band.* The alternative *intensive noun* reading will surface when the same word pattern is combined with a root that describes a “human quality” like {bxl} *stinginess*, {n
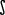
t^
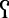
^} *activity* or {ħkm} *wisdom* to give rise to the forms [baxiilun] *extremely stingy*, [na
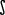
iit^
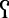
^un] *very active* and [ħakiimun] *very wise* [Fn en0002].^2^ These examples demonstrate that two surface forms may share the same phonological structure, and yet express word patterns with different morphemic properties.

### Verbs

There are 10 verbal word patterns in current use, divided into one unaugmented and nine augmented patterns (Bohas & Guillaume, 1984; El-Dahdah & Matar, 1990; Holes, 1995; Wright, 1995). The unaugmented word pattern can have one of the three following forms: {fa
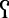
ala}, {fa
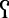
ila}, {fa
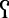
ula} and is typically referred to as Pattern 1, while the remaining nine augmented forms are referred to as Patterns 2–10[Fn en0003].^3^ These word patterns modify the basic grammatical and meaning properties of the root in a highly predictable way. The pattern {fa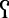 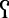
ala}, for example, usually modifies the root meaning by making it *intensive*. Accordingly, the unaugmented surface form [qatala] means *kill*, while the augmented form [qattala] means *massacre*. Similarly, the pattern {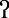
infa
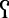
ala} confers a *reflexive* reading on the root such that the unaugmented [xada
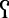
a] means *deceive*, but the augmented [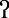
inxada
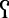
a] means *let oneself be deceived*.

A given word pattern can be associated with more than one syntactic reading. The augmented word pattern {fa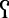 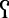
ala}, for example, is frequently associated with an *intensive* reading implying that an action is done with great violence as in [kassara] *break into pieces*, or during a long time as in [t^
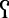
^awwafa] *often go around* (Wright, 1995). But when the same word pattern is combined with the root {kðb} *lying*, the resultant surface form [kaððaba] *belie* is obtained. Here the word pattern carries an *estimative* ^4^ rather than an *intensive* reading, signalling the speaker’s attitude towards the truth and falsity of a given proposition. When this same word pattern is combined with a third type of root – for example with {ktb} *write* to form the phonetic word [kattaba] *cause to write* – its syntactic reading is now *causative*. Note that these variations between estimative, causative, or intensive readings are superimposed on a set of basic morpho-syntactic values which remain unchanged across these variations. Although the pattern {fa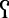 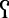
ala} has three different interpretations in the examples above, these interpretations all have in common the core features [+verb, +active, +perfective].

As regards roots, their basic semantic value will be variably expressed in the different verbal surface forms in which they occur. For example, the meaning of *separating* expressed by the root {frq} is encountered in all of the following verb forms: [faraqa] *separate*, [farraqa] *scatter*, [faaraqa] *disengage*, [tafarraqa] *split*, and [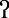
infaraqa] *be divided*. By contrast, the root {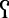
rd^
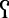
^} expresses the semantic field of *being large* which is present in the surface forms [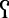
arud^
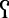
^a] *become wide* and [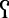
arrad^
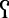
^a] *broaden*. However, this meaning does not surface in other forms like [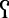
aarad^
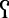
^] *to resist* and [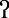
a
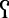
rad^
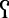
^a] *to be averse.* This demonstrates that the initial semantic meaning of the root may become opaque in some derivations.

### Primitive nouns

Unlike deverbal nouns, which are thought to be directly related to verbs, primitive nouns are widely held to be basic non-derived constructs (Wright, 1995). Primitive nouns can contain three consonants (e.g., [qirdun] *monkey*), four consonants (e.g., [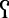
aqrabun] *scorpion*) or five (e.g., [safar
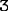
alun] *quince*). The number of word patterns involved does not exceed 19 (Bohas & Guillaume, 1984). Primitive nouns are traditionally analysed as comprising a root and a word pattern in the same way as deverbal nouns and verbs. Accordingly the noun [qirdun] is made up of the root {qrd} and the word pattern {fi
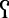
lun}, and the noun [safar
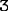
alun] is broken into the root {sfr
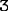
l} and the word pattern {fa
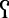
allalun}. However, the contribution of the word pattern to the overall meaning of surface forms in primitive noun morphology is much less constrained than in the deverbal noun morphology. A word pattern like {fa
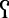
lun} conveys a feminine reading in [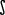
amsun] *sun*, but a masculine reading in [kalbun] *dog*, while the pattern {fu
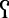
ulun} has a singular reading in {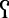
unuqun} *neck*, but a plural one in [suħubun] *clouds*. The productivity of root morphemes in primitive nouns is very limited, typically with only a few commonly used forms, such as a singular and a plural (Wright, 1995).

## Stem-based models

Stem-based approaches to Arabic take many different but related forms (e.g., Benmamoun, 1999, 2003; McCarthy & Prince, 1990; Ratcliffe, 2013). Here we will focus on the imperfective-stem account as developed by Benmamoun (1999, 2003), as a representative of the core claims of such approaches. In this framework, the root and word pattern are dispensed with on the grounds that they play no role either in deriving surface word forms or in conveying semantic and grammatical information. The imperfective stem, the residual core of imperfective verbs when prefixes and affixes are stripped off, takes on the role of both the root and the pattern in supporting the derivation of surface word forms. Accordingly, an imperfective active verb form like [yu
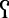
allim] *he teaches* is not parsed into the prefix ya~, the root {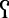
lm} and the word pattern {fa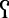 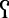
il}, but into the prefix *ya* and the stem [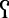
allim]. This stem is then used as the basic building block to derive other forms – for example, by prefixing *mu~* to get [mu
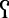
allim] *teacher*, or the prefix 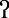
u~ to form the imperfective [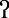
u
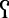
allim] *I teach*.

On this account word pairs that share only a word pattern (e.g., [xuruu
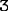
un]-[nuzuulun] *going out-landing*) cannot be considered to be related by virtue of sharing a common underlying linguistic element. In a psycholinguistic context (as in the current research) this makes clear predictions for the outcome of priming experiments, where lexical representations are probed using related and unrelated prime-target pairs. For all prime-target pairs which share an underlying imperfective stem, priming is predicted. For all others, no priming is predicted, and a “covering hypothesis” is required to explain cases where priming is nonetheless obtained.

This covering hypothesis will typically invoke a dimension of the stimulus pairs along which they are nonetheless related, with the natural candidates being phonological and semantic. The plausibility of such accounts is examined below, with Experiment 1 asking whether priming between words sharing the identical phonological structure is obtained when the test pairs are not morphologically related, and Experiments 3 and 4 providing evidence relevant to potential semantic accounts, based on direct semantic overlap between primes and targets.^5^

## Previous research into Semitic morphology

Most studies that have addressed the role of morphemic units in Semitic word recognition have done so within the framework of the root and pattern model, applied both to Hebrew and to Arabic (see Berent, Vaknin, & Marcus, 2007; Vaknin & Shimron, 2011 for examples of stem-based research). Building on earlier work by Feldman, Frost, and Pnini (1995), Frost, Deutsch, Forster, and others have investigated the role of word pattern and root morphemes in Hebrew visual word recognition (e.g., Deutsch, Frost, & Forster, 1998; Deutsch, Frost, Pollatsek, & Rayner, 2000; Frost, Deutsch, Gilboa, Tannenbaum, & Marslen-Wilson, 2000; Frost et al., 1997), while Boudelaa and Marslen-Wilson (e.g., 2004a, 2011, 2013) have pursued related issues in Arabic for both spoken and written words.

The research into Hebrew, almost all using masked priming techniques, gives a consistent but mixed picture of the applicability of the root and pattern approach to the mental representation of Hebrew morphology, and to the decomposability of complex Hebrew surface forms into underlying morphemic components. Three main themes emerge from this research. First, root priming emerges consistently for prime-target pairs sharing a root, whether these are nominal forms such as [tizmoret] *orchestra*, primed by the root {zmr} *singing* (Frost, Forster, & Deutsch, 1997) or verbal forms such as [hilbish] *he dressed* primed by the form [hitlabesh] *he got dressed* (Deutsch et al., 1998) – although more recent research suggests that root priming becomes much weaker for less productive roots (Velan & Frost, 2011).

Second, root priming both in nominal and verbal contexts is not affected by semantic transparency. This holds not only for masked priming – consistent with later masked priming research in English and French (e.g., Longtin, Segui, & Hallé, 2003; Rastle et al., 2000) – but also when an overt cross-modal priming task is used (Frost, Deutsch, Gilboa, Tannenbaum, & Marslen-Wilson, 2000). This contrasts with cross-modal priming in Indo-European, where priming is generally not observed between derivationally related primes and targets sharing a stem when this relationship is semantically opaque (e.g., Marslen-Wilson et al., 1994; but see Smolka, Komlosi, & Rosler, 2009).

Thirdly, research in Hebrew finds consistent differences across both masked and cross-modal priming tasks in the degree of priming between verbal forms sharing a word pattern and nominal forms sharing a word pattern. Frost et al. (1997) found no priming between pairs of Hebrew nouns that share a nominal word pattern – such as [taklIt]/[/targIl][Fn en0006],^6^ *record/exercise*. In contrast, Deutsch et al. (1998) found consistent priming between verb forms sharing a word pattern, as in pairs like [hilbis]-[hikriv], which both contain the verbal word pattern {HI- -I-}. Parallel results emerged in the Frost et al. (2000) cross-modal study, using the same sets of verbal and nominal materials. The consistent failure of priming for Hebrew nominal word patterns suggests that Hebrew deverbal nouns are not fully decompositionally represented, and is potentially consistent with a stem-based rather than a root and pattern approach.

The picture that emerges from research into the processing of Arabic morphological structure, while still incomplete, seems to overlap only partially with the picture for Hebrew. In common with the first two strands seen for Hebrew, Boudelaa and Marslen-Wilson find that Arabic words sharing a root morpheme prime robustly, and regardless of whether they share a transparent or an opaque semantic relationship (e.g., Boudelaa & Marslen-Wilson, 2004a, 2005, 2013). These results hold not only for masked priming tasks selectively tapping early stages of visual word recognition, but also for cross-modal tasks that reflect the properties of central morpho-lexical representations.

The effects for word patterns are much more divergent. As in Hebrew, there is consistent evidence for priming between items sharing a verbal word pattern, across a range of tasks and linguistic populations (e.g., Boudelaa & Marslen-Wilson, 2004a, 2005, 2013). Quite distinct from Hebrew, however, we also find strong priming for words sharing only a nominal word pattern (e.g., Boudelaa & Marslen-Wilson, 2005, 2011). As noted earlier, the deverbal nominal system in Arabic – with more than 500 nominal word patterns – is a substantial and productive component of the Arabic lexical landscape, and a critical domain for evaluating the applicability of stem-based and root and pattern approaches.

In the research reported in this paper, we focus on extending and completing this emerging picture of Arabic as providing the clearest evidence for the active role of abstract morphemic units in the representation and processing of complex spoken and written forms in a Semitic language. The properties of different aspects of the Arabic mental lexicon, as indicated above, are patched together from a variety of different sources, and are incomplete in certain critical respects – most saliently where the evidence for the morphological (as opposed to phonological) status of nominal word pattern priming is concerned. We seek here to give a more cohesive overview of the overall structure of the lexical system of Arabic, the central exemplar of the Semitic language family.

To do so, we will bring together a set of experiments that document the realisation of roots and word patterns across all the major domains of Arabic derivational morphology. The first two studies (Experiments 1 and 2) explore the cognitive status of nominal and verbal word patterns in Arabic, where the linguistic system allows us to directly separate out morphological and form-based factors – a distinction which is critical for evaluating the competing claims of root and pattern as opposed to stem-based approaches. The second set of studies (Experiments 3 and 4) conduct complementary tests, evaluating the relative roles of semantic and morphological factors in root priming (Experiment 3), and the degree of abstractness of the root morpheme representations accessed in the priming process (Experiment 4). These two experiments also provide additional tests for stem-based accounts, since not all forms which share a root necessarily share an imperfective stem. The final study (Experiment 5) extends the examination of word pattern and root priming effects to the hitherto untested primitive noun category unique to Arabic.

## Arabic in context

The version of Arabic that we work with here is known as Modern Standard Arabic (MSA). The descendant of Classical Arabic, MSA is not only the medium of writing in the Arab world, but also dominates the mass media (radio, television, and the press). It is the language of education throughout much of the Arab world and overrides the many vernaculars acquired as a first language by all native Arabs and used in informal settings, and which shares its fundamental properties with these vernaculars (Badawi, 1973; Holes, 1995; Versteegh, 1997). In this situation of linguistic diglossia, there are several reasons why we chose to focus on MSA rather than one of the dialects spoken in the Arab world. At a practical level, the absence of a written form of the Arab dialects not only means the absence of basic lexicographic and statistical information, but also rules out experimental techniques which use written materials. To maintain continuity with research in other languages, and to probe the abstract properties of lexical representations, it is necessary to use tasks like cross-modal priming where the prime is auditory and the target is written (or, indeed, masked priming where both prime and target are written).

Second, MSA begins to be learnt very early, almost in parallel with the spoken regional dialect. Arab children are now exposed to MSA from infancy through the electronic mass media (radio and television), are likely to use it exclusively throughout the educational process, and are acquainted with the basics of writing MSA as early as age 3 or 4 in kindergartens. Third, there are major parallels between the structure of MSA and of any given dialect of Arabic. Where morphology is concerned, both MSA and dialectal Arabic are Semitic systems in which roots and word patterns dominate word formation (Badawi, 1973; Holes, 1995; Versteegh, 1997). In other research we have specifically addressed potential processing differences in the morphological domain between MSA and dialectal Arabic (as spoken in southern Tunisia). Using an auditory-auditory priming task, we established that standard and dialectal Arabic exhibited very similar facilitatory priming patterns (Boudelaa & Marslen-Wilson, 2013) with no differences in speed or accuracy of responses. Not only did MSA and the spoken dialect exhibit parallel psycholinguistic structural properties, but also there was no sign of differences in familiarity or fluency in processing either variety.

### Experiment 1: nominal word patterns in Arabic

The first two experiments deal with the word pattern morpheme, focusing in Experiment 1 on the deverbal nominal system. It is this that presents the most theoretically critical contrast with Hebrew, as well as a strong test of stem-based approaches. These contrasts and tests depend, however, on convincing evidence that nominal word pattern priming is indeed morphological in nature, and not primarily phonologically (or even semantically) driven. Arabic nominal word patterns offer the possibility of such a test, since some of them are apparent homophones, with the same phonological structure associated with two distinct morpho-syntactic roles. This makes it possible to directly contrast phonological and morphological factors, while neutralising possible semantic effects. A word pattern like {fu
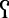
uulun} can have the two readings *deverbal noun*, *singular*, *masculine* or *plural noun*. This allows us to co-vary morphemic overlap in prime-target pairs, while holding phonological overlap constant. In Condition 1 of Experiment 1, the word pattern has the same morpho-syntactic role in both prime and target (see Table 1). The target word [ħuduuθun], *happening*, a deverbal masculine singular noun, is primed by [xud^
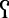
^uu
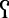
un], *submission*, also a deverbal masculine singular noun, where both incorporate the word pattern {fu
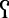
uulun}.

**Table 1.** Experiment 1: design, sample stimuli (with Arabic script, IPA transcription, and English glosses), and relevant prime statistics by condition: average number of letters and syllables, prime root productivity (Root Prod), and rated familiarity.

	Prime	Target	Letters	Syllables	Root Prod	Familiarity
1. [+WP +F +M]	خضوع [xud^ 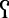 ^uu 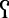 un]*submission*	حدوث[ħuduuθun]*happening*	4.13 (0.34)	3.13 (0.34)	16.79 (7.18)	3.78 (0.25)
2. [+WP +F –M]	سجون [su 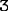 uunun]*prisons*	حدوث[ħuduuθun]*happening*	4.13 (0.34)	3.13 (0.34)	16.08 (9.92)	3.79 (0.23)
3. [+Phonology]	اتحاد[ 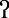 ittiħaadun]*union*	حدوث[ħuduuθun]*happening*	4.50 (0.59)	3.54 (0.59)	16.79 (9.35)	3.74 (0.22)
4. [Unrelated]	استاذ [ 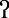 ustaaðun]*teacher*	حدوث[ħuduuθun]*happening*	4.50 (0.59)	3.130.34	14.63 (7.47)	3.76 (0.19)

The nominal word patterns used here are chosen to be productive and transparent. We selected seven nominal word patterns^7^ which apply in a systematic manner to a broad range of Arabic verbal roots with predictable morpho-syntactic consequences. For example, the “deverbal noun, singular, masculine” reading of the word pattern {fu
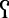
uulun} will typically apply when it is combined with a root morpheme that expresses either physical movement, as in the roots {nzl} *landing* or {xr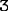}, *going out*, or more abstract movement, as in {ħdθ} *happening* (i.e., transition from one state to another).

In Condition 2, the same set of nominal target words was paired with prime words whose word patterns, while phonologically identical in terms of their CV structure and vocalic properties, diverged morpho-syntactically. Most of the prime words were drawn from within the deverbal noun system, but had word patterns with different morpho-syntactic functions – exemplified in the contrast between {fu
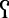
uulun} indicating *deverbal singular masculine noun* and {fu
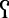
uulun} indicating *plurality* (as in the prime word [su
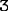
uunun], *prisons*). A small number of prime words were drawn from the set of primitive nouns, where the word patterns generally do not have a systematic morpho-syntactic interpretation. If priming between words sharing word patterns depends on them sharing the same morpheme, defined as a grammatical as well as a phonological entity, then we should not see priming for these pairs. But if priming through shared word patterns is a function of shared phonological similarity, independent of its possible linguistic interpretation, then we should see priming for these pairs.

Performance in Conditions 1 and 2 is compared to a phonological control in Condition 3, where prime and target have consonantal overlap like [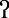
ittiħħaadun]/[ħuduuθun] *union/happening*, but do not share either a word pattern or a root morpheme, and to an unrelated baseline condition like [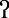
ustaaðun]/[ħuduuθun] *teacher/happening*.^8^

The stem-based approach predicts no facilitation based on a shared word pattern since this is not a unit of lexical representation. If facilitation is found, this will need to be attributed to other properties of the relationship between prime and target. The current experiment provides a control for a potential phonological similarity account, by virtue both of the Condition 1/Condition 2 contrasts and of Condition 3. A direct or mediated semantic priming account is excluded because the conditions for successful cross-modal priming on this basis – in particular, the presence of strong semantic relatedness – are not present in the +WP pairs in Conditions 1 and 2.

To test these predictions, we used the cross-modal priming task, where an auditory prime is immediately followed by a visual target, rather than the masked priming task predominantly used by Frost and colleagues. This is because the focus of the theoretical and empirical questions here is on the properties of central representations of morpho-lexical knowledge. By using an overt priming task and by switching input modality between prime and target, we prevent responses being driven primarily by early morpho-orthographic parsing (e.g., Rastle & Davis, 2008), and therefore failing to reflect the properties of more central representations (Marslen-Wilson, Bozic, & Randall , 2008). Note, however, that a striking property of both Hebrew (Frost et al., 2000) and Arabic (e.g., Boudelaa & Marslen-Wilson, 2004b) is that masked and overt priming tasks do not seem to deliver different answers.

A second reason is that masked priming may not be optimal for detecting primarily phonological effects (Holyk & Pexman, 2004; Rastle & Brysbaert, 2006). This is a disadvantage when one of the goals of the experiment is to test for a potential phonological basis to word pattern priming. Thirdly, the use of cross-modal priming maintains continuity with previous work on languages like English, Italian, and German that has used the same task (e.g., Clahsen & Fleischhauer, 2014; Marslen-Wilson et al., 1994; Orsolini & Marslen-Wilson, 1997; Sonnenstuhl, Eisenbeiss, & Clahsen, 1999).

### Method

#### Preliminary tests

To prepare the experimental materials to be used in the five experiments reported here, we ran two preliminary tests to estimate the subjective familiarity of a large set of words and the semantic relatedness between them.

Twelve hundred words were chosen for the semantic relatedness test. The participants in these tests were mainly Tunisian and Moroccan students from the University of Paris. Fifteen of them (average age 26) were asked to rate each pair on a 9-point scale ranging from *not related at all* (1) to *highly related* (9). Another 15 subjects from the same population took part in the familiarity judgement test. They were presented with the 1200 words and instructed to rate them on a 5-point scale with 1 being least familiar and 5 most familiar. In both tests, the written instructions prompted the subjects to respond within three seconds and to move on to the following item if they encountered an unknown word. We calculated familiarity for each word and estimated semantic relatedness for each pair by averaging the ratings across subjects. Only words having an average rating of 3 or more in the familiarity test were selected for further use in the priming experiments. The semantic relatedness values for the relevant conditions are reported in the individual Materials and Design section for each experiment, with semantically related prime-target pairs averaging a rating value of 6 (SD: 0.8), and semantically unrelated pairs averaging 2.5 (SD: 0.5). Only semantically unrelated pairs were used in Experiments 1 and 2.

#### Participants

A group of 40 volunteers, aged 16–20, took part in Experiment 1. They were pupils at the High School of Tataouine in southern Tunisia and were fluent users of MSA. They had been exposed to MSA from early childhood via the electronic mass media, and were educated in MSA from entry into primary school at age six. None of them had any history of hearing loss or speech disorder.

#### Materials and design

Twenty-four orthographically unambiguous deverbal nouns were selected for use as priming targets. The full list of experimental materials is provided in an appendix. Each target was paired with four different primes in a multi-condition within-item design to generate four experimental conditions each with 24 sets of prime-target pairs (see Table 1). The targets were 4–5 letters long (SD: 0.34) and averaged 3.13 syllables (SD: 0.34) with an average familiarity of 3.75 (SD: 0.23) (see Appendix 1). Table 1 also lists the relevant psycholinguistic properties of the primes in each condition, including length in letters, length in syllables, root productivity (defined as the number of words formed by a given root), and rated subjective familiarity. The principal test conditions (1, 2, and 4) were closely matched for length, but the restrictions on choice of stimuli in Condition 3 (phonological control), where primes and targets needed to phonologically overlap but without sharing potential roots or word patterns, led to slightly longer primes (see Table 1). This is reflected in significant differences across conditions in letter (*F*(3,92) = 4.871, *p* < 0.001) and syllable length, (*F*(3,92) = 6.053, *p* < 0.001). We address these length mismatches across conditions using analyses of covariance.

In preparing these visually presented target materials, additional constraints were imposed by the properties of Arabic orthography. As noted above, the Arabic script represents consonants and long vowels by letters, while short vowels are marked by diacritics (similar to the system of “pointing” in Hebrew script). In normal texts intended for adult readers, the diacritics are omitted, leading to potential ambiguities in the reading of many forms, since different short vowels may occur with the same orthographically specified CV frame. To minimise problems of homography and ambiguity, we chose the visual targets to be as orthographically unambiguous as possible, primarily through the use of words containing long vowels.

As a further check that the visual targets were read with the intended word pattern, matching as appropriate the word pattern in the auditory prime, we ran a test on the target words of Experiments 1 and 2 by intermixing them with another 48 non-ambiguous words ranging between 3 and 7 letters long, and presenting them to a group of 30 subjects in an offline naming task. Fifteen of the 30 subjects were asked to read each word aloud as fast as they could while the first author noted the reading that was produced. The subjects read almost every word in the way we intended them to be encoded as targets in the two Experiments[Fn en0009].^9^ The other group of 15 participants were asked to read the 48 targets intermixed with the same 48 target fillers as in the first test. However, this time each target was presented at the offset of its associated auditory prime (as used in the priming experiment), while the fillers were preceded by unrelated auditory primes. The visual targets were displayed for 5 seconds and the participant had to read them aloud as quickly as possible while the first author noted down any words that were not produced as anticipated. The presentation of the primes and targets was self-paced using the Superlab software (Cedrus, Phoenix, Arizona). In this offline naming task, every participant read each word exactly as we intended them to, using the same word pattern (phonologically) as the auditory prime.

In Condition 1, the label [+WP +F +M] refers to primes and targets sharing word patterns matched for form and morpho-syntactic properties, and where the morpho-syntactic role of the word pattern in this condition is systematic and productive. None of the prime-target pairs share a semantic relationship, as standardly defined (average relatedness of 1.71). The full morphemic match between word patterns in Condition 1 contrasts with Condition 2, where the label [+WP +F –M] refers to prime and target pairs sharing the phonological structure of the word pattern but not the same morpho-syntactic meaning. The word patterns in the primes and targets in Condition 2 typically had strongly diverging syntactic properties. The semantic relatedness of the prime-target pairs in Condition 2 was again low, averaging 1.38.

Condition 3, labelled [+Phonology], provides an additional control for phonological overlap, where prime and target also overlap in form, but there is no potential morphemic relationship between them. Since the form overlap between prime and target in Conditions 1 and 2 is non-linear, in the sense that the prime and target pairs do not overlap in any two consecutive phonemes, primes and targets in Condition 3 also share a non-linear form overlap as illustrated by the pair [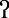
ittiħaadun]/[ħuduuθun] *union/happening*, which are semantically and morphologically unrelated. Here the overlap relates to the underlined consonants /ħ, d/.^10^ A standard unrelated baseline for Conditions 1–3 is provided by Condition 4 [Unrelated] where prime and target have no semantic, morphological, or phonological properties in common.

Condition 3, labelled [+Phonology], provides an additional control for phonological overlap, where prime and target also overlap in form, but there is no potential morphemic relationship between them. Since the form overlap between prime and target in Conditions 1 and 2 is non-linear, in the sense that the prime and target pairs do not overlap in any two consecutive phonemes, primes and targets in Condition 3 also share a non-linear form overlap as illustrated by the pair [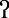
ittiħaadun]/[ħuduuθun] *union/happening*, which are semantically and morphologically unrelated. Here the overlap relates to the underlined consonants/ħ, d/.^10^ A standard unrelated baseline for Conditions 1–3 is provided by Condition 4 [Unrelated] where prime and target have no semantic, morphological, or phonological properties in common.

Test-pairs from the four conditions were rotated across four experimental lists, such that each target word only occurred once in each list. The overall proportion of relatedness was reduced by including 46 unrelated prime-target filler pairs which were matched with the experimental pairs on familiarity and form class. The proportion of related items in each list was less than 30%, treating the [+WP +F +M], [+WP +F –M] and [+Phonology] conditions as related. A further 70 words were selected and paired with pseudowords so as to reflect the form overlap between the word-word pairs. For example, the target [huruubun] *running away* is paired with the pseudoword prime *[fuxuudun] which consists of the word pattern {fu
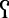
uulun} and a non-existing root created by changing one or two letters of an existing root. We also included catch trials to ensure that participants attended to both the auditory prime and to the visual target. In these catch trials, participants were prompted, immediately after they had responded to the visual target, to write down the auditory stimulus they had just heard. There were 24 of these trials (12 word-word and 12 word-non-word), distributed pseudo-randomly across the stimulus list, and always followed by a non-test prime-target pair. Practice trials comprised 20 prime-target pairs with 10 word responses and 10 pseudoword responses. Each of the 4 experimental lists contained 184 pairs of which 92 were word-word pairs and 92 word-pseudoword pairs.

#### Procedure

All the prime words were recorded by a native speaker of Arabic and digitised at a sampling rate of 44 kHz, then downsampled to 22 kHz using the CoolEdit program and stored on a portable PC (Dell, Inspiron 7000). Two portable PC monitors were used to test subjects in pairs in a quiet room. They heard the stimuli at a comfortable level through HD 250 Sennheiser headphones. The sequence of stimulus events within each trial was as follows: An auditory prime was presented and immediately at its offset a target was displayed on the screen for 2000 ms. A new trial would start at the end of this period unless the subject responded within the time-out. Timing and response collection were controlled by the PC running the DMDX package. Subjects were instructed to make a lexical decision as quickly and as accurately as possible by pressing a “YES” or “NO” key. The “YES” response was assigned to the dominant hand. The experiment, which lasted about 12–15 minutes, started with the 20 practice trials followed by the rest of the stimuli.

### Results

The mean reaction times (RTs), standard deviation, and error rate for each of the experimental conditions are shown in Table 2, together with the main priming effects. In the RT analysis, decision latencies which were more than two standard deviations below or above the mean of each subject were excluded. The 0.3% of data excluded by this constraint was not replaced. A further 3.7% of the data was excluded from the RT analyses due to response errors (misses and false positives).This procedure was followed in all the experiments reported here.

**Table 2.** Experiment 1: mean lexical decision times (standard deviations in parentheses), priming effects (relative to Condition 4), and % error.

Condition	RT (ms)	Priming (ms)	% Error
1. [+WP +F +M]	602 (58)	33	5
2. [+WP +F −M]	639 (72)	−4	2
3. [+Phonology]	660 (81)	−25	5
4. [Unrelated]	635 (67)		4

In this and the following experiments the RT data were submitted to a two-way ANOVAs, on participants and on items, combined to compute the min-*F*’ statistic, with the two four-level factors of priming condition and experimental list.^11^ We also performed a by-items univariate ANCOVA with priming condition as a fixed factor and number of syllables^12^ as a covariate to evaluate the possible effects of the longer primes in the [+Phonology] condition on the overall pattern of results.

The RT analyses revealed a significant main effect of priming condition by participants and items (*F*_1_(3,39) = 7.99 *p* < .001; *F*_2_(3,23) = 5.29 *p* <.003; min-*F*’ (3,50) = 3.18, *p* = .03). No interaction was found between priming condition and list (*F*_1_ < 1, *F*_2_ < 1)_._ The ANCOVA revealed similar results with a significant main effect of priming condition (*F*_2_(3,23) = 4.63 *p* =.02).

Planned comparisons revealed a significant 33 ms difference between Condition 1 [+WP +F +M] and the [Unrelated] baseline condition (*t*_1_(39) = 2.85, *p* =.007; *t*_2_(23) = 2.39, *p* =.025). Condition 1 also differed significantly both from Condition 2 [+WP +F −M] (*t*_1_(39) = 3.44, *p* < .001; *t*_2_(23) = 3.40, *p* =.002), where prime target share the phonological form but not the morpho-syntactic function of the word pattern, and from the [+Phonology] control condition (*t*_1_(39) = 4.27, *p* < .001; *t*_2_(23) = 4.20, *p* < .001). In contrast, the difference between Condition 2 [+WP +F −M] and the [Unrelated] condition was not significant (*t*_1_ < 1, *t*_2_ < 1), nor was the difference between Condition 2 and the [+Phonology] control condition (*t*_1_(39) = 1.82, *p* > .05; *t*_2_(23) < 1). The interference effect of 25 ms for the [+Phonology] condition, relative to the [Unrelated] baseline, was significant (*t*_1_(39) = 2.19, *p* < .034; *t*_2_(23) = 2.07, *p* < .049).

The error data were submitted to mixed-effects logistic regression analyses using R: A Language and Environment for Statistical Computing (R Core Team, 2014) and the R packages glmer. In each of the five experiments, error rate was modelled as a function of Condition with subjects, prime words, target words, and rotation as random variables.These mixed-effects logistic regression analyses of the error rates revealed no significant effects.

### Discussion

Experiment 1 demonstrates that word pattern priming effects for Arabic deverbal nouns can be found when the prime and target share both the phonological structure of the word pattern and its core morpho-syntactic function. Pairs like [xud^
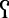
^uu
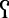
un]/[ħuduuθun] *submission/happening* and [ni
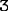
aaratun]/[ħilaaqatun] *carpentry/hairdressing* facilitate each other by virtue of sharing the phonological structure and the morpho-syntactic content of their respective word patterns {fu
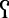
uulun} and {fi
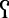
aalatun}. In contrast, when prime and target share the phonological structure of the word pattern but not their core morpho-syntactic content, as in pairs like [su
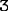
uunun]/[ħuduuθun], then no priming occurs. Word patterns like {fu
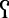
uulun} seem to be genuine homophones, with phonological patterns which correspond to two different underlying morphemes.

This interpretative framework is necessarily rejected by a stem-based account where the word pattern is not recognised as an independent property of lexical representation in Arabic. It must therefore look for a covering hypothesis to explain the patterns of priming across the conditions of Experiment 1. However, a further salient feature of the results is that the priming observed in Condition 1 between pairs of deverbal nouns sharing a word pattern is not amenable either to an explanation in terms of semantic relatedness or to a purely form-based account, in terms of phonological overlap. Where semantic factors are concerned, prime-target pairs sharing only a word pattern are rated as being semantically unrelated, offering no basis for a semantic interpretation of the priming effects (whether direct or mediated via a linking third representation).

The phonological account is ruled out by the results for Condition 2, where the degree of phonological overlap is exactly comparable to the overlap in Condition 1 but no priming is obtained. In Condition 3, which provides a different kind of control for possible form effects, we also see no facilitatory priming. Here the prime-target pairs exhibited a non-linear form overlap, as exemplified by the pair [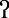
ittiħaadun]/[ħuduuθun] *union/happening*, which share the consonantal letters /ħ, d/. Far from facilitating responses to the target, this partial consonantal overlap gives rise to significant interference effects. Consonantal material in Arabic is typically part of the root morpheme, and carries semantic information. The interference here, therefore, may be due to competition between different root morphemes that are each partially activated in the prime and target (cf., Allen & Badecker, 1999). This is in keeping with cross-modal priming results in a concatenative morphology such as Italian. Phonological overlap in pairs like “volò/voluto” *he flew/he wanted*, where the stem {vol~} is homophonic between the verbs “volare” *to fly* and “volere” *to want*, leads to significant interference in responses to the target “voluto” (Orsolini & Marslen-Wilson, 1997).

Finally, the results confirm the contrast with Hebrew, where no priming is found between nouns sharing a word pattern, both in masked priming studies (Frost et al., 1997) and in studies using the same cross-modal paradigm as here (Frost et al., 2000). While we cannot rule out possible differences in the types of the materials used and in the productivity of the morphemes involved, the reduced online decomposability of Hebrew nouns is likely to reflect a significant cross-linguistic difference between Arabic and Hebrew. Stem-based accounts may achieve more traction in Hebrew, in the absence of evidence for a role of the word pattern in Hebrew deverbal nouns, while the same is not true for Arabic.

### Experiment 2: word patterns in Arabic verbs

This experiment was designed to complement Experiment 1, in the context of our overall questions about cross-linguistic generality and about the status of word patterns as abstract psycholinguistic entities. We know from earlier studies (e.g., Boudelaa & Marslen-Wilson, 2005) that Arabic verbal word patterns, like Hebrew verb patterns, show significant priming. The goal of Experiment 2, in addition to providing a replication of this result in the context of this general empirical overview of the Arabic mental lexicon, is to explore in more detail what properties determine underlying morphemic identity in verbal word patterns in Arabic, in analogy to the questions asked about nominal word patterns in Experiment 2.

Condition 1 here parallels Condition 1 in Experiment 1, using prime and target pairs sharing a verbal word pattern at the level of both phonology and morphology, as in the pair [ħat^
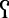
^t^
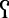
^ama]/[farraqa] *demolish/scatter*, where the word pattern {fa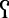 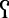
ala} conveys an *intensive* reading in both surface forms (Wright, 1995), over and above its basic syntactic value as *active* and *imperfective*. These word patterns are highly systematic, and far more productive than their counterparts in the nominal morphology. Given the results in Experiment 1, as well as earlier research, there is no reason not to expect priming.

It is not possible, however, to construct a direct analogue of Condition 2 in the previous experiment. The restricted domain of verbal word patterns (with only 10 in current use) does not contain homophones of the type we exploited for the deverbal noun morphology. A verbal word pattern such as {fa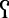 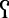
ala} does not have a counterpart which fills an entirely different morpho-syntactic role. The verbal morphology offers more fine-grained contrasts, where a word pattern such as {fa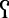 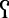
ala} can have an *intensive* reading, as above, when combined with the root {ħt^
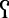
^m} to form the word [ħat^
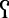
^t^
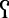
^ama] *to demolish*, but has an *estimative* reading when combined with the root {kðb} to form the word [kaððaba] *to belie*. This is not the same kind of major morpho-syntactic contrast that we used in Experiment 1. The forms [ħat^
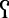
^t^
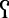
^ama] and [kaððaba] are both verbs, with their primary morpho-syntactic properties held in common.

It is an open question whether these more fine-grained variations in the linguistic functions of Arabic verbal word patterns (despite their salience in traditional linguistic accounts of Arabic) will disrupt priming to the same extent as the contrasts in Experiment 1. We explained the absence of priming for noun-noun pairs such as [su
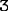
uunun]/[ħuduuθun] on the basis that they did not share the same underlying word-pattern morpheme. If the word pattern {fa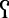 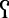
ala}, when used to generate an intensive verb form, does not invoke the same underlying morpheme as {fa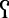 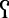
ala} when it is used to generate an estimative word form, then we should not expect to see priming in Condition 2. If, however, as the close morpho-syntactic similarities underlying these different uses of {fa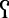 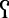
ala} might suggest, the underlying morpheme is the same in all these cases, then priming will be preserved. Either way, the outcome will shed light on the functional specificity of morphemic representations in Arabic.

Finally, as in Experiment 1, we include a phonological overlap condition and an unrelated baseline condition.

### Method

#### Participants

Forty high school volunteers from the same linguistic background and age group as before took part in Experiment 2.

#### Materials and design

The target words were 24 verb forms spanning the nine augmented word patterns. As in Experiment 1, the visually presented targets were selected to be orthographically unambiguous. The selected targets were on average 4.46 letters long (SD: 0.93), 3.13 syllables (SD: 0.34) with an average root productivity of 26.21 (SD: 11.73), and a familiarity of 3.93 (SD: 0.15) (see Appendix 2). To form four experimental conditions, each of these targets was paired with four prime types, as shown in Table 3. As before, Table 3 also lists the relevant prime distributional statistics. A series of one-way ANOVAs established that the four priming conditions did not differ significantly along any of these dimensions (all *F*’s < 1).

**Table 3.** Experiment 2: design, sample stimuli (with Arabic script, IPA transcription, and English glosses) and relevant prime statistics by condition: average number of letters and syllables, prime root productivity (Root Prod), and rated familiarity.

	Prime	Target	Letters	Syllables	Root Prod	Familiarity
1. [+WP +F +M]:	حطّم [ħat^ 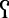 ^t^ 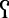 ^ama]*demolish*	فرّق[farraqa]*scatter*	4.46 (0.93)	3.54 (0.51)	17.67 (8.19)	3.89 (0.18)
2. [+WP +F −M]:	كذّب [kaððaba]*belie*	فرّق[farraqa]*scatter*	4.46 (0.93)	3.54 (0.51)	17.00 (7.49)	3.91 (0.19)
3. [+Phonology]:	مرق[maraqun]*broth*	فرّق[farraqa]*scatter*	4.96 (1.46)	3.54 (0.83)	16.46 (8.87)	3.89 (0.21)
4. [Unrelated]:	لعب [la 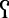 ibun]*playing*	فرّق[farraqa]*scatter*	4.29 (0.91)	3.46 (0.83)	16.67 (8.64)	3.92 (0.22)

As in Experiment 1, the first condition, labelled [+WP +F +M], contains pairs which share both the phonological structure and the full morpho-syntactic function of the word pattern. For example, the pair [ħat^
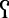
^t^
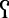
^ama]/[farraqa] *demolish/scatter* share Pattern 2, [fa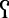 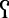
ala], which has an intensive reading in both forms. In condition 2, labelled [+WP +F −M], the prime and target pairs share the phonological structure of the word pattern but not its full morphosyntactic role. The target [farraqa] *demolish*, for example, is now paired with [kaððaba] *belie*, a prime in which the word pattern {fa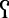 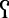
ala} has an estimative rather than an intensive reading. Semantic relatedness was low in both Conditions 1 and 2, averaging 2.04 and 1.54, respectively.

In Condition 3, labelled [+Phonology], prime and target pairs share a non-linear form overlap but have no morphological (or semantic) relationship. As in Experiment 1, these are pairs that have two consonants in common. In Condition 4, labelled [Unrelated], primes and targets share no linguistic or meaning relationship. Note that the prime words in Conditions 3 and 4 are nominal forms instead of verb forms. This was necessary to avoid the vocalic overlap that would otherwise have existed between primes and targets if we had used verb forms as primes. According to multilinear phonological theory, Arabic vowels can function as independent morphemic units, so that a vocalic overlap between primes and targets could produce an unwanted additional morphological relationship (Hoberman, 1988; McCarthy, 1981; Wright, 1995).

The proportion of relatedness in each of the four experimental lists, the nature and the number of the fillers, the word-pseudoword pairs, and the training set were similar to those used in Experiment 1.

#### Procedure

The procedure was identical to the previous experiment.

### Results

In this experiment, 3.75% of the data were false positives and misses. The data cleaning procedure removed another 0.5% of the data, which were not replaced. Mean RTs, standard deviations, and error rates are displayed in Table 4, together with the priming effects for the relevant conditions. RTs were subjected to ANOVAs with participants (*F*_1_) and items (*F*_2_) as random factors, while error rates were analysed using mixed-effects logistic regression. The analysis of the RT results began with a two-way ANOVA with the two four-level factors of priming condition and experimental list.

**Table 4.** Experiment 2: mean lexical decision times (standard deviations in parentheses), priming effects (relative to Condition 4), and % error.

Condition	RT (ms)	Priming (ms)	% Error
1. [+WP +F +M]:	644 (70)	36	5
2. [+WP +F −M]:	641 (77)	39	3
3. [+Phonology]:	691 (119)	−11	4
4. [Unrelated]:	680 (100)		5

There was a significant main effect of priming both by participants and items (*F*_1_(3,39) = 6.48 *p* < .001; *F*_2_(3,23) = 3.74 *p* = .016; min*-F*’ (3,48) = 2.37, *p* = .08). The interaction between priming condition and experimental list did not reach significance either in participants or in items analyses (*F*_1_ and *F*_2_ < 1). Planned comparisons using a paired sample *t*-test showed that Condition 1 [+WP +F +M] was significantly different both from the baseline condition [Unrelated], with (*t*_1_(39) = 2.93, *p* = .006; *t*_2_(23) = 2.84, *p* = .048), and from the [+Phonology] control condition, with (*t*_1_(39) = 2.50, *p* = .018; *t*_2_(23) = 2.57, *p* = .017). However, the difference between Condition 1 [+WP +F +M] and Condition 2 [+WP +F −M], where the word patterns shared by prime and target diverge in their semantic interpretation, was not significant (*t*_1_ < 1 and *t*_2_ < 1), with the two conditions showing the same amount of priming, at 36 and 39 ms, respectively. Condition 2 was significantly different from both the [+Phonology] control condition, with (*t*_1_(39) = 3.36 *p* = .002; *t*_2_(23) = 2.79, *p* = .01), and the [Unrelated] condition, with (*t*_1_(39) = 3.59 *p* < .001; *t*_2_(23) = 1.93, *p* = .065). The 11 ms difference between the phonological control condition and the unrelated condition did not approach significance (*t*_1_ < 1, *t*_2_ < 1).

The mixed-effects analyses of error rates showed no significant effects across experimental conditions.

### Discussion

The results of Experiment 2 are clear. There is effective priming in Condition 1, between primes and targets sharing word patterns that have the same phonological properties and the same morpho-syntactic functions. This result, which is comparable to the effects in Condition 1 of Experiment 1, and consistent with the results for Hebrew verb patterns, confirms that abstract morphemic entities, which never appear as independent surface phonetic forms, can function as active linguistic and cognitive elements in the processing and representation of Arabic verbs.

The equally strong priming in Condition 2 was less predictable, since the word patterns in prime and target diverge significantly in the meaning classes into which the resulting words fall. The results indicate that, nonetheless, the same underlying grammatical morpheme is being activated in both prime and target – as much so, it seems, as in Condition 1. The word pattern [fa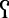 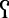
al], for example, as Pattern II of the nine augmented verbal word patterns, seems to constitute the same underlying linguistic and cognitive entity irrespective of whether it combines with a root to form a causative, intensive, or estimative verb. This suggests that these different meaning classes, though salient in traditional linguistic analyses of verbal word patterns, do not reflect major underlying differences at the morphemic level, which may be structured in terms of basic grammatical functions such as voice and aspect.

It is implausible that the results for Condition 2 can be accounted for on the basis of phonological overlap. Although prime and target share considerable phonological material, the results for the same condition in Experiment 1 show that this by itself is not enough to guarantee priming. Phonological overlap needs to be accompanied by morphological overlap in order for priming to occur. Furthermore, in Condition 3, where primes and target only overlap in their phonological form, there is no sign of facilitation, and a tendency towards interference.

To summarise, the first two experiments confirm that word patterns in Arabic show significant priming, not only for the verbal morphology, as in Hebrew, but also for the deverbal noun morphology. In both experiments, an account in terms of either phonological or semantic overlap between prime and target seems ruled out. This cluster of results is problematic for a stem-based account (Bat-El, 2003; Benmamoun, 2003; Berent et al., 2007). The prime-target pairs we used (e.g., [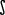
uruu
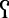
un]-[duxuulun] *starting*-*entry* in Experiment 1, and [farraħa]-[qat^
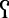
^t^
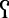
^a
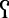
a] *make happy*-*cut into pieces* in Experiment 2) cannot be said to be morphologically related on a stem-based account; at best such pairs would be classed as phonologically related. But the results for Condition 2 in Experiment 1, as well as for the phonological control conditions in both experiments, seem to rule out an analysis simply in terms of phonological overlap. The consistent facilitation we observe for prime and target pairs sharing a word pattern morpheme, under these carefully controlled phonological conditions, seems incompatible with a stem-based psycholinguistic model of the Arabic mental lexicon.

We now turn to an examination of the other principal component of the Arabic word, the consonantal root morpheme.

### Experiment 3: root priming and semantics

The investigation of the Arabic consonantal root in the next two experiments addresses two sets of questions. First, complementing the preceding experiments on the word pattern morpheme, we address similar issues about the generality of claims about the properties of morphemes in the Semitic mental lexicon. The existence of word pattern priming, as documented in Experiments 1 and 2, presupposes an analysis process which separates information about the word pattern of a surface form from information about its root. It is therefore no surprise that we also see robust priming between Arabic words sharing a root (e.g., Boudelaa & Marslen-Wilson, 2005).

A second, theoretically critical issue, bearing on the general question of how morphological and semantic factors interact to determine the properties of lexical representations, is the finding that roots show priming irrespective of the semantic transparency of the surface word form. In English, we see a strong interdependence between transparency and apparent morphological decomposition and priming in overt priming tasks (Marslen-Wilson et al., 1994). Synchronically opaque words like *department*, where the current meaning of the whole form is not related to the meaning of the stem *depart*, do not show stem-based priming. Research in Polish, another language with a concatenative morphology, shows similar results, with strong effects of semantic transparency on morphological decomposability (Reid & Marslen-Wilson, 2000).

Patterns of this sort led to a view of morphological structure where, although distinguishable from semantic factors, there is a close relationship between them. Complex words were argued to be lexically represented in morphologically decomposed format only when this delivered the right semantic outcome. This in turn leads to an emphasis on the role of compositional semantics in the construction of lexical meaning, so that transparent complex forms like *darkness* were assumed not to have stored meanings but with their meaning being computed, as required, by combining the meanings of the stem *dark* with the affix *ness*.

The results reported for Hebrew and Arabic fly in the face of these assumptions. Several experiments consistently show robust root priming between prime/target pairs where at least one of the words involved is semantically opaque (e.g., Boudelaa & Marslen-Wilson, 2005, 2013; Deutsch et al., 1998; Frost, Kugler, & Forster, 2005; Frost et al., 1997), so that the meaning of the surface form cannot be computed by combining the meanings of the root and the word pattern. This is inconsistent with any universal claim that morphological decomposition in the mental lexicon is dependent on semantic interpretability, and also undermines the view that morphological decomposability (in central lexical representations) goes hand-in-hand with online computation of meaning.

Experiment 3, accordingly, focuses on semantic variables in relation to the presence or absence of root priming, using items from the deverbal noun morphology as primes and targets. Condition 1 provides the baseline root priming condition, using semantically related prime/target pairs such as [madxalun]/[duxuulun] *inlet/entry* and [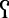
at^
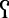
^uufun]/[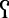
at^
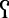
^fun] *compassionate/compassion*, where each pair shares a root, and where the meaning of the root is semantically transparent throughout. Note that semantic transparency here is defined, along similar lines to work in Indo-European languages, as the overt judgement that the meaning of the whole form is related to the predominant semantic value conveyed by the root in question. Thus, in the prime/target pair [madxalun] *inlet* and [duxuulun] *entry*, the standard meaning of the root {dxl} as *entering*, *coming in* is transparently expressed in both forms, as reflected in semantic relatedness judgements in native speakers. For both prime and target, the root is combined with a nominal word pattern (respectively {maf
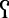
alun}, with the morphosyntactic reading *place noun*, and {fu
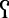
uulun} with the reading *deverbal noun singular*), to produce prime-target pairs falling within the deverbal nominal domain of the Arabic lexicon.

This condition is labelled [+Root +S], and contrasts with Condition 2, [+Root −S], where the same targets are preceded by prime words which share the same root, but where the meaning of the word is not transparently related to the standard meaning of the root – as in [mudaaxalatun]/[duxuulun] *conference/entry*, and [mi
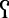
t^
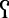
^afun]/[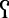
at^
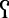
^fun] *coat/compassion*. We expect nonetheless that strong root priming should still be observed.

From a stem-based perspective, the prime-target pairs used here do not share an imperfective stem as standardly defined, despite the fact that they share a root. This makes it uncertain what are the psycholinguistic predictions of the stem-based approach in these specific priming contexts. For the examples mentioned above ([madxalun]/[duxuulun] *inlet/entry* and [mudaaxalatun]/[duxuulun] *conference/entry*), there are different stems attributable to each form. While the stem in a word like [madxalun] *inlet* can be analysed (as in Benmamoun, 2003) as the CCVC-structure {dxal}, by the same token the stem of [duxuulun] *entry* is analysed as {dxuul} and that of [mudaaxalatun] *conference* as {daaxal}. This raises problems for predictions of priming, since neither of these prime-target pairs directly share an imperfective stem, necessary to provide a common representational basis for priming. While it may be possible, in linguistic terms, to construct a derivational process whereby all these forms are linked to a common underlying abstract stem, this would require a mediated priming process which has not been found to be effective in lexical decision tasks (e.g., Balota & Lorch, 1986; Shelton & Martin, 1992).

We also include two further comparison conditions. Condition 3 [−Root +S] evaluates the effects of semantic relatedness between prime/target pairs when they do not share a root, as in pairs like [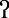
iilaa
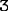
un]/[duxuulun] *insertion/entry* and [raħmatun]/[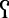
at^
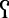
^fun] *mercy/compassion*. Pure semantic priming in cross-modal priming is typically weaker and less robust than morphologically based effects. A final baseline condition, labelled [Unrelated], presents the same targets preceded by primes to which they are neither semantically, morphologically, nor phonologically related.

We did not include a further phonological control condition, mimicking the form overlap between primes and targets sharing a root. This was because the comparable conditions in Experiments 1 and 2, labelled [+Phonology], where primes and targets shared two or more consonants, but no morphological relationship, showed no sign of any facilitatory effects. This makes it implausible that any priming between items that do share a root can be attributed simply to form overlap.

Conditions 2 and 3 serve a further role here in evaluating the viability of stem-based accounts, by allowing us to measure the potential strength of semantic factors in generating facilitatory priming between primes and targets. To explain priming where primes and targets do not share linguistic units recognised by stem-based approaches, these approaches can seek covering hypotheses based on either phonological or semantic confounds. Experiments 1 and 2 exclude a phonological account but only indirectly exclude a semantic account, since this dimension was not explicitly manipulated. Here we can ask (1) whether priming is still obtained when there is no semantic link (as in Condition 2) and (2) what is the strength of semantic priming when there is no accompanying shared morphological link. The answers to both these questions will bear on the plausibility of attempts to preserve stem-based accounts through semantically based covering hypotheses.

### Method

#### Participants

Forty volunteers from the same age group and linguistic background as the previous studies took part in this experiment.

#### Materials and design

The targets were 24 non-ambiguous deverbal nouns with average letter length of 4.13 (SD: 0.34), syllable length of 3.17 (SD: 0.38), root productivity 24.04 (SD: 8.35), and familiarity of 3.92 (SD: 0.22) (see Appendix 3). These were paired with 96 prime words, also formed with the use of nominal word patterns. The prime/target pairs fall into four experimental conditions, using a within-item design such that the same target occurs with four different types of prime. The characteristics of the primes in terms of length in letters and syllables, root productivity, and familiarity are given in Table 5. The primes differed across conditions only in number of syllables *F*(3,92) = 2.91, *p* = 0.038, and the effects of this variable will be addressed in an ANCOVA.

**Table 5.** Experiment 3: design, sample stimuli (with Arabic script, IPA transcription, and English glosses) and relevant prime statistics by condition: average number of letters and syllables, root productivity (Root Prod), and rated familiarity.

	Prime	Target	Letters	Syllables	Root Prod	Familiarity
1. [+Root +S]:	ممتع [mumti 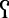 un]*enjoyable*	متعة [mut 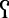 atun]*pleasure*	4.63(0.77)	3.46(0.66)	24.04 (8.35)	3.89(0.21)
2. [+Root −S]:	متاع [mataa 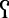 un]*commodity*	متعة [mut 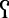 atun]*pleasure*	4.88(0.90)	3.71(0.69)	24.04 (8.35)	3.93(0.23)
3. [−Root +S]:	لذّة [laððatun]*enjoyment*	متعة [mut 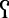 atun]*pleasure*	4.17(0.87)	3.42(0.78)	21.46 (7.66)	3.90(0.24)
4. [Unrelated]:	خراج [xaraa 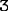 un]*tax*	متعة [mut 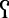 atun]*pleasure*	4.50(0.59)	3.17(0.38)	20.46 (12.46)	3.88(0.28)

The label [+Root +S] in the first condition refers to primes and targets sharing a root and a transparent semantic relationship. Such pairs are judged to be semantically highly related by the 15 judges who took part in the pre-test, with an average value of 8.25. In the second condition, labelled [+Root −S], the prime and target share a root morpheme, but their semantic overlap is opaque. This is reflected in much lower relatedness ratings, of 3.08. Condition 3, [−Root +S], contains pairs which do not share a root but are highly semantically related (average relatedness of 7.54). Priming in these first three conditions is measured against a baseline condition in which the target is paired with an unrelated prime. In none of the four conditions do the prime and target share a word pattern morpheme.

The proportion of related prime/target pairs was reduced by including 46 unrelated prime-target filler pairs, controlled for familiarity and form class. A further 70 word-pseudoword pairs were constructed so as to mimic the overlap between the experimental pairs. The number of catch trials and practice trials, as well as the overall number of trials per list, was the same as in the previous experiments. No vowel diacritics were used and all the target words were orthographically unambiguous.

#### Procedure

The same procedure as in Experiment 1 was used.

### Results

The data pruning procedure applied as before resulted in the removal of no data points. The percentage of errors due to false positives and misses was 4.25%. Table 6 presents the mean RTs, error rates, and priming effects for the three related conditions relative to the unrelated condition. As in the previous experiments, correct decision time latencies were analysed using a two-way ANOVA, by participants (*F*_1_) and items (*F*_2_), with the four-level factor of priming condition and the second factor of experimental list. Additionally, a one-way ANCOVA was performed using priming condition as a fixed factor and syllable length as a covariate to evaluate possible effects of differences in syllable length between conditions.

**Table 6.** Experiment 3: mean lexical decision times (standard deviations in parentheses), priming effects (relative to Condition 4), and % error.

Condition	RT (ms)	Priming (ms)	% Error
1. [+Root +S]:	549 (73)	54	4
2. [+Root −S]:	552 (75)	51	4
3. [−Root +S]:	582 (89)	21	5
4. [Unrelated]:	603 (88)		4

There was a significant main effect of priming condition (*F*_1_(3,39) = 9.93 *p* < .001; *F*_2_(3,23) = 6.56 *p* < .001, min*-F*’ (3,50) = 3.95, *p* = .01) and no interaction with experimental list (*F*_1_, *F*_2_ < 1). The main effect of priming condition was similarly significant in the ANCOVA taking into account variations in prime syllable length (*F*_2_(3,23) = 5.32 *p* = .01). Planned comparisons showed that the [+Root +S] condition was significantly different from the [Unrelated] condition (*t*_1_(39) = 4.28, *p* = .01; *t*_2_(23) = 3.83, *p* = .01), with a mean priming effect of 54 ms. Comparable effects were observed for the [+Root −S] condition, with 51 ms of priming relative to the [Unrelated] condition (*t*_1_(39) = 4.10, *p* = .01; *t*_2_(23) = 3.65, *p* = .01). In contrast, the effects in the [−Root +S] condition, where there was no morphological relation between prime and target, were very much weaker (at 21 ms) and not significantly different from the [Unrelated] condition (*t*_1_, *t*_2_ < 1). Priming effects in the two root priming conditions, [+Root +S] and [+Root −S], were significantly stronger than in the [−Root +S] condition (*t*_1_(39) = 2.97, *p* = .006; *t*_2_(23) = 2.86, *p* = .007) and (*t*_1_(39) = 2.34, *p* = .024; *t*_2_(23) = 2.30, *p* = .026), respectively.

Finally, the analysis of error rates using mixed-effects logistic regression revealed no significant effects.

### Discussion

The results give an unequivocal answer to the two questions asked in this experiment. They confirm that prime-target pairs of deverbal nouns sharing a root morpheme prime strongly, and that they do so irrespective of the semantic transparency of the relationship between them. Priming is as strong for [+Root −S] pairs as it is for [+Root +S] pairs. This complements the results for Experiments 1 and 2, where a morphological unit that is non-semantic in nature, the word pattern, also gives reliable priming effects.

We found no additional advantage for the presence of a semantic link between prime and target, even though Frost et al. (2000) did find a small but significant advantage cross-modally in Hebrew. Generally, however, semantic effects in cross-modal priming can be weak and labile (e.g., Moss & Marslen-Wilson, 1993). This also seems to be the case here, as reflected in the [−Root +S] Condition, where a semantic relation alone did not elicit significant priming. Both of these results for the semantic dimension substantially weaken the plausibility of accounts of priming in the [+Root] conditions (or in the [+WP] conditions in Experiments 1 and 2) in terms of semantic confounds rather than relationships between root and word pattern morphemes shared by primes and targets.

Overall, these results confirm the generality, for the Semitic language family, of priming between pairs of nouns or pairs of verbs sharing a root. The Arabic tri-consonantal root morpheme, despite its discontinuous realisation in the surface phonetic string, is separated out as a functionally distinct element during the processing of spoken and written deverbal nouns. The irrelevance of semantic factors to the presence (or strength) of root effects suggests that the underlying processes are intrinsically morphological in nature.

### Experiment 4: root priming across nouns and verbs

We can probe in more detail the properties of underlying root morphemes – in particular, the abstractness of the cognitive entities involved – by looking for root priming across morpho-syntactic categories. Arabic verb morphology includes both verbs proper and deverbal nouns. The syntactic distinction between verbs and deverbal nouns is signalled by differences in the word patterns applied in the two domains, not by differences in the roots involved. This implies that we should still obtain root priming when the prime is a deverbal noun and the target is a verb, as long as they share the same root, and as long as the same underlying cognitive entity is accessed for both nominal and verbal readings of this root.

Because of the close morphological relationship between verbs and deverbal nouns in Arabic, there are thousands of pairs such as [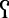
aqlun] *mind* and [ta
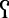
aqqala] *be mindful*, which share the same root but which fall into different syntactic categories because of the word patterns with which they are combined. In root and pattern terms, the deverbal noun [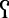
aqlun], meaning *mind*, results from the combination of the root {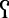
ql} with the deverbal word pattern {fa
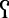
lun}, which typically has the reading *noun, singular*. The verb [ta
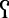
aqqala] *be mindful*, in contrast, results from the combination of the root with a verbal word pattern, in this case {tafa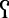 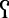
ala}, with the morpho-syntactic reading *perfective, active, effective* [Fn en0013] *.* ^13^ Both prime and target are semantically transparent, and we expect to see priming between them.

In contrast, from the point of view of a stem-based approach, it is again equivocal whether priming should be predicted between pairs like [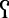
aqlun]/[ta
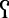
aqqala] *mind/be mindful* and [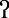
i
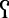
tiqaalun]/[ta
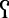
aqqala] *imprisonment/be mindful*. These forms have different stems – {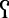
aql}, {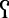
aqqal} and {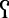
tiqaal}, respectively. Even if these stems could be related to the original CCVC-stem {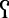
qal} under appropriate derivational assumptions, their relationships will be too remote, with too many mediating items (Boudelaa, 2014). For instance, the link between [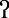
i
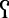
tiqaalun] and [ta
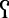
aqqala] cannot be direct since they have different stems, it has to be mediated by [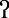
i
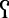
taqala] *arrest*, and [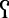
aqala] *tie*. Under such circumstances little or no priming can be expected.

The [+Root +S] condition is contrasted, as in Experiment 3, with [+Root −S] pairs like [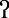
i
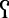
tiqaalun]/[ta
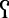
aqqala] *imprisonment/be mindful*. These pairs also do not share a stem, but they share a root and fall into different morphological categories as a result of the word pattern that the root combines with. However, the prime words (always deverbal nouns) are now semantically opaque. If morphological decomposition into roots and word patterns is not dependent on semantic factors, and if the same underlying root is participating in both deverbal nouns and verbs, then the root and pattern approach will again predict priming.

We also include, as in Experiment 3, a further [−Root +S] comparison condition, where test pairs have a transparent semantic relationship but no morphological relationship, as well as the standard baseline condition.

### Method

#### Participants

We tested 40 volunteers from the same linguistic background and age group as in the previous experiments.

#### Materials and design

Twenty-four surface verb forms, averaging 4.38 letters (SD: 0.92) and 3.36 syllables in length (SD: 0.49), and 25.71 in root productivity (SD: 10.59), and 3.88 in familiarity (SD: 0.28) were selected for use as targets (see Appendix 4). In all of these forms the verb citation reading was by far the most dominant reading. Each target was paired with four deverbal nouns yielding the experimental design shown in Table 7, together with the average statistics for length in letters and syllables, root productivity and familiarity. The priming words did not differ significantly across conditions on these variables (all *F*’s < 1).

**Table 7.** Experiment 4: design, sample stimuli (with Arabic script, IPA transcription, and English glosses), and relevant prime statistics by condition: average number of letters and syllables, and rated familiarity.

	Prime	Target	Letters	Syllables	Root Prod	Familiarity
1. [+Root +S]	عقل [ 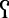 aqlun]*mind*	تعقّل [ta 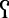 aqqala]*be mindful*	4.42(0.78)	3.79(0.72)	25.71 (10.59)	3.90(0.30)
2. [+Root −S]	اعتقال [ 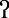 i 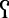 tiqaalun]*imprisonment*	تعقّل [ta 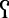 aqqala]*be mindful*	4.50(0.66)	3.58(0.72)	25.71 (10.58)	3.91(0.29)
3. [−Root +S]	رزانة[razaanatun]*mindfulness*	تعقّل [ta 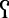 aqqala]*be mindful*	4.38(0.97)	3.33(0.92)	24.33 (9.49)	3.85(0.23)
4. [Unrelated]	عافية [ 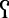 aafiyatun]*well being*	تعقّل [ta 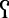 aqqala]*be mindful*	4.25(0.90)	3.50(0.83)	24.29 (12.63)	3.87(0.29)

Paralleling Experiment 3, Conditions 1–3 varied morphological and semantic relations between primes and targets. For all conditions, semantic relatedness is based on the pre-tests described earlier, with average values of 7.42 for Condition 1, 3.08 for Condition 2, and 6.88 for Condition 3. In Condition 1 [+Root +S] a deverbal noun primes a verb form target with which it shares a root morpheme and a transparent semantic relationship. In Condition 2 [+Root −S] prime and target again have a root in common, but the prime is semantically opaque. In Condition 3 [−Root +S] the prime and target are semantically highly related but do not share a root (or word pattern). Condition 4 [Unrelated] provides the baseline for assessing priming effects in the other three conditions.

The proportion of related prime/target pairs in each of the four experimental lists, the nature and the number of the fillers, the word-pseudoword pairs of the catch trials and the training set were the same as in the previous experiments.

#### Procedure

The procedure was identical to the previous experiments.

### Results

Mean RTs, priming effects, and error rates are displayed in Table 8. Our standard pruning procedure removed no subjects or items. The overall percent error due to false alarms and misses was 4.75%.

**Table 8.** Experiment 4: mean lexical decision times (standard deviations in parentheses), priming effects (relative to Condition 4), and % error.

Condition	RT (ms)	Priming (ms)	% Error
1. [+Root +S]:	550 (76)	56	5
2. [+Root −S]:	566 (71)	40	5
3. [−Root +S]:	581 (90)	25	4
4. [Unrelated]:	606 (86)		5

Reaction-times were subjected to two-way ANOVAS on subjects and items, with priming condition and experimental list as the two four-level factors. The main effect of priming condition was significant both by subjects and items (*F*_1_(3,39) = 9.95 *p* < .001, *F*_2_(3,23) = 9.65 *p* = .016; min*-F*’ (3,57) = 4.90 *p* = .004). The interaction between priming condition and experimental list was not significant (*F*_1_ and *F*_2_ < 1). Paired sample comparisons confirmed a significant 56 ms difference between the [+Root +S] condition and the [Unrelated] condition (*t*_1_(39) = 5.47, *p* < .001; *t*_2_(23) = 4.65, *p* < .001). The [+Root +S] condition also differed from the [−Root +S] condition (*t*_1_(39) = 3.71, *p* = .01; *t*_2_(23) = 3.26, *p* = .03). Turning to Condition 2 [+Root−S], there was a significant 40 ms effect relative to the [Unrelated] condition (*t*_1_(39) = 4.38 *p* = .01; *t*_2_(23) = 3.93, *p* = .01). The size of the priming effects in the two [+Root] conditions (1 and 2) differed significantly by subjects (*t*_1_(39) = 2.08, *p* = .044); but not by items (*t*_2_(23) = 1.15, *p* > 0.25)[Fn en0014].^14^ Compared with the [−Root +S] condition, the [+Root −S] priming effect also differed significantly by subjects (*t*_1_(39) = 2.72, *p* = .011) but not by items (*t*_2_(23) = 1.70, *p* = .1). The effects in the [−Root+S] condition, where there was no morphological relation between prime and target did not approach significance (*t*_1_ < 1 and *t*_2_ < 1).

The analysis of errors using mixed-effects logistic regression yielded no significant results.

### Discussion

The results are again clear. There is robust priming between primes and targets sharing a root, even when the two words come from different syntactic categories, and irrespective of the semantic transparency of the items involved. Priming is just as strong in Condition 1, at 56 ms, as it was in the comparable condition in Experiment 3, where prime and target came from the same grammatical category. Although priming is reduced to 40 ms in the semantically opaque Condition 2, this is of doubtful importance given that the difference with Condition 1 is not significant by items (see also footnote^15^) and that the effect of morphological relatedness remains statistically robust.

More generally, these results underline the abstractness of the cognitive entities that support root priming in Arabic, based on a relationship between shared morphemic elements at a level of representation that feeds into both the verbal and the nominal morphology. They also confirm the independence of root priming effects in Arabic from the semantic properties of the words involved.

In contrast, these results are less straightforward for stem-based models to account for, since the prime-target pairs used here do not share the same stem, despite sharing a root. For a pair like [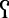
aqlun]/[ta
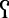
aqqala], their respective stems are {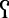
aql} and {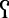
aqqal}, which could only prime morphologically through a mediating derivational sequence linking them to a shared parent morpheme. Alternatively, such an account might try to accommodate priming between the [+R +S] pairs by positing lexical links between semantically related words, but this fails to explain the preservation of priming in the [+R −S] pairs and is undermined by the weakness of priming for the [−R +S] pairs.

We now turn to a final experiment examining the remaining component of Arabic morphology, the set of primitive nouns.

### Experiment 5: roots and word patterns in primitive nouns

Primitive nouns in Arabic are a set of words covering historically basic concepts, and differ in several respects from the set of deverbal nouns – these, as we noted above, derive from verbal roots in a productive and open morphological framework. Primitive nouns, in contrast, do not derive from verbal roots, and form a closed set of about 120 members in current usage. Although primitive nouns are standardly analysed into roots and word patterns, these morphemic components are more opaque and less productive than the corresponding elements in the verbal morphology and its derivatives. This allows us to assess the importance of these variables in Arabic morphemic organisation, as reflected in dynamic priming effects.

Nineteen different word patterns are used to form primitive nouns. These are limited in the range of meanings they convey, and are often inconsistent from one form to the next. The same word pattern may convey a singular reading combined with one root, as in [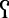
unuqun] *neck*, but a plural reading when combined with a different root, as in [kutub] *books*. In addition, the productivity of these word patterns is low, with many of them applying to no more than four or five roots. In contrast, the verbal word patterns potentially combine with hundreds of roots, and many of the deverbal noun word patterns are also very productive. Note that some of the primitive noun word patterns are homophones, in the sense that they have the same phonological structure as other, more productive word patterns in the deverbal noun morphology. However, so long as these homophonous word patterns are not the same morphemes in terms of their underlying morpho-syntactic function, then they should be representationally distinct in the Arabic mental lexicon, as the results of Experiment 1 indicate.

Turning to the primitive noun roots, these are transparent in meaning, but are typically not productive. Many roots only appear in singular and plural forms, a few others also have diminutive and adjectival forms – the root {qrd}, for example, from the noun [qirdun] *monkey*, combines with the word pattern {fu
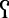
uulun} to form the plural [quruudun] *monkeys*, and with the word pattern {fu
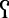
eilun} to form the diminutive noun [qureidun] *small monkey*. Verbal roots, in contrast, not only combine with several verbal word patterns, but also with many nominal word patterns, to form deverbal nouns.

In the context of the four preceding experiments, these properties of primitive nouns raise several questions about the role of their constituent morphemes in access and representation, and whether word pattern and root priming effects will be obtained. In Experiment 1, we obtained priming between deverbal nouns sharing a word pattern, so long as the word patterns were morphemically as well phonologically equivalent. Given the variability of the primitive noun word patterns, as well as their much lower productivity, it is unclear whether priming is to be expected here. Where roots are concerned, the low productivity of the primitive noun roots may affect their salience as organising units in lexical access and representation, with possible consequences for priming effects between items sharing roots. Experiment 5, accordingly, investigates both word pattern and root priming effects for primitive nouns.

The design of this experiment is restricted by the small set of available items, and by the lack of variation along certain dimensions. There are very few examples of forms which are semantically opaque, ruling out the use of a [+Root −S] condition as in Experiments 3 and 4. Instead, we focus on roots and word patterns, and include a phonological control condition (as in Experiments 1 and 2) rather than a semantic control condition – Experiments 3 and 4 already provide a sufficient estimate of the strength of purely semantic priming effects in Arabic cross-modal priming tasks.

Condition 1 [+Root +S] pairs primes and targets with the same roots, and where each member of the pair is semantically transparent. Reflecting the distributional properties of the primitive noun set, two-thirds of the pairs are singular-plural pairs, as in [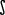
umuusun]/[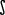
amsun] *suns/sun* or [dafaatirun]/[daftarun] *copy-books/copy-book*. Note that these are always Arabic “broken plurals”, where the surface form of the plural is highly divergent from the form of the singular (Boudelaa & Gaskell, 2002; McCarthy & Prince, 1990; Ratcliffe, 1998). This is unlike both the suffixing “sound plurals” found in other components of the Arabic system, and the regular suffixing plurals found in languages like French or English. The effect of this is that, just as in Experiments 3 and 4, the appearance of priming depends on the ability to disentangle the root from its phonological context, rather than matching surface strings in the prime and target. A remaining third of the test pairs covered a range of grammatical relations, including the diminutive, as in [ðu
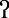
eibun]/[ði
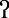
bun] *small wolf/wolf*, and relational adjectives, as in [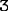
abaliyyun]/[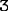
abalun] *mountainous/mountain*.

Condition 2, looking for word-pattern priming, is similar to Condition 1 in Experiment 1, and is labelled [−Root +WP]. Here the same target words, in a within-word design, are primed by words with which they share only a word pattern. The word [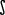
amsun] *sun*, for example, shares the word pattern {fa
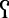
lun} with the prime [baɤlun] *mule*. The word pattern has the same morpho-syntactic function in both words, with the interpretation *singular noun*. Similarly, in the prime-target pair [bas^
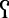
^alun]/[mat^
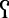
^arun] *onion/rain*, the word patterns share the reading mass noun. In Experiment 1, the deverbal noun word patterns sharing the same morpho-syntactic function showed significant priming. However, as noted above, these deverbal noun patterns are more productive and more consistent than the primitive noun patterns, and this may affect priming.

Finally, the experiment also included two further conditions – a phonological control condition, as in Experiments 1 and 2, and an unrelated baseline condition. Following the earlier results, we expect the [+Phonology] condition, where there is a non-linear phonological overlap – as in pairs like [samiidun]/[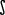
amsun] *semolina/sun* – to show no priming, and possibly some interference.

### Method

#### Participants

Forty volunteers from the same age group and linguistic background as in the previous experiments were tested.

#### Materials and design

The material consisted of 24 target primitive nouns (see Appendix 5) which consisted on average of 3.21 (SD: 0.41) letters, 2.54 (SD: 0.51) syllables, with root productivity of 7.46 (SD: 5.44), and familiarity of 3.93 (SD: 0.26). These targets were paired with four sets of 24 prime words as shown in Table 9 which gives length in letters and syllables and average root productivity and familiarity for each condition. The primes did not differ across conditions in root productivity and familiarity (all *F*’s < 1), but they did on length in letters *F*(3,92) = 29.72, *p* < .001, and syllables *F*(3,92) = 13.34, *p* < .001. These differences reflect the difficulties in matching on length when working with the restricted primitive noun stimulus pool. We use an ANCOVA analysis to evaluate the possible effects of these length differences on priming.

**Table 9.** Experiment 5: design, sample stimuli (with Arabic script, IPA transcription, and English glosses), and relevant prime statistics by condition: average number of letters and syllables, prime root productivity (Root Prod), and rated familiarity.

	Prime	Target	Letters	Syllables	Root Prod	Familiarity
1. [+Root +S]	شموس [ 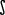 umuusun]*suns*	شمس [ 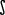 amsun]*sun*	4.54(0.51)	3.29(0.46)	7.46(5.44)	3.89(0.22)
2. [−Root +WP]	عنز [ 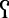 anzun]*goat*	شمس [ 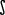 amsun]*sun*	3.21(0.41)	2.54(0.51)	7.88(7.21)	3.91(0.18)
3. [+Phonology]	سميد [samiidun]*semolina*	شمس [ 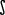 amsun]*sun*	4.17(0.64)	3.21(0.41)	7.71(4.88)	3.90(0.29)
4. [Unrelated]	ناقة [naaqahun]*she-camel*	شمس [ 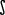 amsun]*sun*	4.42(0.58)	3.29(0.55)	7.92(3.80)	3.92(0.25)

In Condition 1, labelled [+Root +S], the prime and target share the same root but do not share a word pattern. Sixteen of these pairs were broken plural/singular sets, five were relational/singular pairs, two were diminutive/singular pairs, and one was a masculine/feminine pair ([kalbatun]/[kalbun] *bitch/dog*). Semantic relatedness ratings for these test pairs averaged 8.08. This is comparable to the 8.24 value for the [+Root +S] deverbal noun pairs used in Experiment 3. Unlike the verbal roots, which normally have three consonants, primitive nouns include roots with four or even five consonants. In the materials used here, 20 had three-consonant roots and 4 had four consonants.

In Condition 2, labelled [−Root +WP], the prime and target share the word pattern but not the root. For 16 pairs, prime and target shared a primitive noun word pattern with the interpretation *singular noun*, as in [baɤlun]/[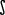
amsun] *mule/sun*, while 8 pairs shared a word pattern with the interpretation *mass noun*, as in [maraqun]/[labanun] *sauce/milk*. As in previous word pattern contrasts, semantic relatedness was low, averaging 1.42.

Condition 3, labelled [+Phonology], includes prime-target pairs sharing a non-linear form overlap whereby they have two to three segments in common, but have no systematic morphological or semantic relationship. Condition 4, labelled [Unrelated], provides a baseline for Conditions 1–3, with primes and targets that are entirely unrelated.

Forty-six unrelated prime-target filler pairs, matched for familiarity and form class, were selected to dilute the overall proportion of related pairs. A further 70 words were selected and paired with non-words. Half of these word-non-word pairs shared form overlap and half did not, matching the filler word-word pairs. The number of catch trials (24) and of practice trials (20) was the same as in the previous experiments. Pseudowords were constructed by combining a non-existent root with an existing word pattern.

Four experimental lists were constructed, each containing 184 pairs of which 92 were word-word pairs and 92 word-pseudoword pairs. The proportion of related items in each list was less than 30%. Subjects were assigned randomly to one of the lists and were not presented with the same prime or target more than once. The stimuli were rotated within conditions in each list in a Latin-square design. Although none of the target words contained vowel diacritics, they were phonologically unambiguous.

#### Procedure

This was the same as in the previous experiments.

### Results

Mean decision time latencies for correct responses in the four experimental conditions and error rates (7%) are reported in Table 10. The same pruning procedure applied to the previous experiments was applied here and did not exclude any data points.

**Table 10.** Experiment 5: mean lexical decision times (standard deviations in parentheses), priming effects (relative to Condition 4), and % error.

Condition	RT (ms)	Priming (ms)	% Error
1. [+Root +S]:	550 (65)	51	7
2. [−Root +WP]:	622 (93)	−21	8
3. [+Phonology]:	626 (96)	−25	5
4. [Unrelated]:	601 (56)		8

The RTs were subjected to two-way analyses of variance, on subjects and on items, with the two four-level factors of prime condition and of experimental list, as in the preceding studies. There was a strong main effect of priming condition (*F*_1_(3,39) = 16.73 *p* < .001, *F*_2_(3,23) = 9.93 *p* = .005, min*-F*’ (3,48) = 6.24 *p* < .001), and no interaction with list. This main effect reflects the facilitatory priming effect for Condition 1 [+Root +S] of 53 ms, and the inhibitory effects of 23 ms and 24 ms, respectively, in the [−Root +WP] and the [+Phonology] conditions. A one-way ANCOVA with priming condition as a fixed factor and number of syllables as a covariate revealed a significant main effect of priming condition (*F*_2_(3,23) = 7.41 *p* = .02).

Further planned comparisons showed the priming effects in the [+Root +S] condition to differ significantly from both the word pattern condition [−Root +WP] (*t*_1_(39) = 5.71, *p* < .001; *t*_2_(23) = 5.55, *p* < .001), the [+Phonology] condition (*t*_1_(39) = 5.20, *p* < .001; *t*_2_(23) = 4.71, *p* < .001, and the [Unrelated] condition (*t*_1_(39) = 6.04, *p* < .001; *t*_2_(23) = 4.96, *p* < .001). There were no other significant effects, with neither the [−Root +WP] condition or the [+Phonology] conditions differing significantly from the baseline [Unrelated] condition, with *t*_1_, *t*_2_ < 1 throughout.

The mixed-effects logistic regression fitted to the error rates did not reveal any significant differences between conditions.

### Discussion

The results of this experiment show both parallels and differences with the results for the verbal and deverbal morphology. First, as in Experiments 3 and 4, we see strong priming for prime/target pairs sharing a root. We interpret this, as before, as reflecting decompositional processes whereby the underlying root morpheme shared by prime and target is accessed both when hearing the prime and when responding to the visually presented target. The size of the resulting priming effect, averaging 51 ms, is comparable to the effects in the four [+Root] conditions in Experiments 3 and 4, which also average 50 ms. This makes it unlikely that the effects here can be attributed to semantic rather than morphological factors. When primes and targets share only a semantic relationship, as in the [−Root +S] conditions in Experiments 3 and 4, the level of priming is much weaker, averaging 23 ms, and is not significant. Furthermore, the degree of semantic relatedness, although high, is no different from the level in the comparable conditions in Experiments 3 and 4.

The primitive noun word patterns, in contrast, show no signs of priming, and behave similarly to Condition 2 in Experiment 1. Unlike these prime/target pairs in Experiment 1, however, which were chosen to dissociate their morpho-syntactic functions, the prime/target pairs here generally share the same overall morpho-syntactic function (either singular noun or mass noun). The fact that nonetheless they show no priming may reflect the lower productivity of primitive noun word patterns (averaging less than 8 in this experiment) relative to their counterparts in the preceding verbal and nominal morphology experiments (where root productivity averaged between 15 and 25), as well as a lack of systematicity in their morpho-syntactic interpretation. Recent research indicates that root productivity is an important determinant of word-pattern priming (Boudelaa & Marslen-Wilson, 2011), and the productivity of the primitive noun roots used here falls into the same range as the low root productivity denominal primes that failed to elicit word pattern priming in the Boudelaa and Marslen-Wilson (2011) study.

## General discussion

We consider here the implications of this research for the cross-linguistic generality of the claims made about the Semitic mental lexicon, for the cognitive status of word patterns in Arabic, and for the role of semantic and phonological factors in priming between morphologically related words in Arabic. In doing so, we will comment on the implications of these outcomes for the viability of stem-based approaches as an account of the psycholinguistic organisation of the Arabic mental lexicon.

### Similarities and differences in the Semitic mental lexicon

A principal goal of this research was to examine the generality of the claims about lexical organisation that have emerged over the past 15 years from experimental psycholinguistic research in Hebrew and in Arabic. Despite substantial divergences where nominal word patterns are concerned (to which we return below), the results we present here for Arabic converge strongly with the work on Hebrew, to establish the pervasive role of morphology as an abstract organising principle in the Semitic mental lexicon.

The most striking contrast with more familiar concatenative morphological systems is the robustness of morphological effects in the face of semantic variation, at all levels of the psycholinguistic system. In English or French derivational morphology, for example, morphological effects in semantically opaque forms are chiefly seen at early stages of lexical analysis, and do not seem to reflect the properties of central lexical representations. While decompositional effects have been reported for opaque forms in German (e.g., Smolka et al., 2009), these seem restricted to verbs with separable affixes, for which specific representational requirements are likely to apply. For Arabic (and, where applicable, for Hebrew) it seems to be the case, across the board, that the morphological structure of underlying lexical representations is as salient for opaque forms as it is for transparently compositional forms. Not only is there no significant modulation of morphological priming effects as a function of semantic relatedness, but this holds true in overt priming tasks just as much as in masked priming.

The second important feature that emerges is the unequivocal abstractness of the cognitive entities that we are dealing with. This applies both perceptually, in the sense that morphemes in Arabic (and Hebrew) are not directly given in the sensory input, and linguistically, in that they apply over major lexical categories. The non-concatenative and discontinuous nature of Semitic word formation in Arabic and Hebrew means that roots and word patterns are not delivered as isolable perceptual units in the phonological or orthographic input. Instead they must be internally reconstructed on the basis of their distributional characteristics. Furthermore, given that Arabic script is primarily consonantal in nature, only incomplete information is usually given in written forms about the underlying word patterns, so that their identity must be inferred on the basis of partial cues.

Arabic and Hebrew are also congruent in terms of the abstract linguistic roles assumed by root morphemes (though see comments in footnote 15). These must be represented at a level of abstraction which allows their deployment across different lexical domains (nominal and verbal morphological systems). For Arabic, as Experiments 3 and 4 showed, priming between roots is as strong across domains as within domains. A root that is combined with a verbal word pattern will prime a target root equally well whether it is combined with a nominal or a verbal word pattern. Similar cross-domain priming for roots is reportedly also seen in Hebrew (Deutsch et al., 1998).

The parallels between the two languages begin to break down, however, when we consider word pattern morphemes. Arabic and Hebrew seem to have similar properties where verbal word patterns are concerned. In both languages, these constitute small, highly productive morphemic paradigms where there is robust evidence for priming between verb forms sharing the same word pattern. There are striking divergences, however, where the nominal word patterns are concerned. Studies in Hebrew, using both masked and overt priming tasks, have consistently shown no priming between nouns sharing only a word pattern. In the Deutsch et al. (1998) model of the Hebrew mental lexicon this is captured by assuming that nominal word patterns do not play a role in the representation and the access of Hebrew nouns – instead, these are linked as whole forms to their roots in a satellite-style format. For verb forms, in contrast, both roots and word patterns are extracted as part of the access process, and the word-level representation of verbs is decompositionally accessed by separable word pattern and root pathways.

This analysis of Hebrew verbal and nominal morphology substantially limits the scope of morphological processes in the representation and processing of words in Hebrew. Many (but not all)^16^ nouns in the language can be linguistically analysed as morphologically complex, made up of a productive root and a word pattern, but this complexity is not fully reflected in the cognitive representation and processing of these forms. In particular, the word pattern does not seem to be directly extracted as part of the recognition process, and its presence and its identity may only become available through post-lexical and metalinguistic processes – as, for example, in the Feldman, Frost, and Pnini (1995) segment-shifting task. In this respect the representation of Hebrew nominal forms may be functionally equivalent to the stem-based representations proposed by some authors (e.g., Berent et al., 2007).

Arabic, by the same logic, is fully decompositional for both nominal and verbal forms. The strong priming between nominal word patterns seen in Experiments 1 and 2 demonstrates the early extraction of both root and word pattern morphemes in the perceptual processing of Arabic nouns. If we were to transpose the Deutsch et al. (1998) model of Hebrew to Arabic, this would result in a simplified model with a uniform treatment of both verbs and nouns. Lexical access for all complex verbal and nominal forms would be mediated by parallel access routes to the word level via separate word pattern and root pathways, where root morphemes are common to both nominal and verbal domains, explaining the ready priming between roots across domains. More generally, this suggests that the core properties of a hypothesised decompositional and morphologically structured “Semitic mental lexicon”, based around discontinuously realised root and word pattern morphemes, are more uniformly exemplified in modern Arabic than in modern Hebrew.

### Form, meaning, and morphological priming

We now turn to more general issues concerning the separability of words and morphemes in psycholinguistic research into lexical representation and processing, with particular reference in Arabic to the competing claims of root and pattern models and stem-based models. We have seen throughout that stem-based models do not provide a direct account of priming patterns in Arabic, because there is little or no evidence that imperfective stems shared by primes and targets are the source of the priming observed in the numerous experiments reported here. This means that stem-based psycholinguistic accounts of lexical representation must establish covering hypotheses, in terms of phonological or semantic overlap between primes and targets, rather than in terms of shared abstract morphemes (roots and word patterns).

The results reported here, and in earlier research, demonstrate, to the contrary, that the dominant feature of word-based priming in Arabic is neither form-based nor semantically based relatedness, but whether or not the prime and target share a morpheme. Form overlap between prime and target (whether phonological or orthographic) generally does not create facilitation, and often produces interference effects. Semantic overlap on its own seems to generate only weak and unstable effects. In contrast, priming between words sharing a morpheme (whether a root or a word pattern) is consistently more robust, and independent of semantic transparency or relatedness.

Direct evidence against phonological accounts of morphological priming in Arabic is provided by the absence of word pattern priming in Condition 2 of Experiment 1. Here the amount of phonological overlap is identical in Conditions 1 and 2, since the primes and targets in both cases share the same word pattern, viewed as a phonological structure, but with the difference that in Condition 1 the shared word patterns are underlyingly the same morpheme, while in Condition 2 they are different morphemes (see Table 1). The priming outcome here is inconsistent with the claim that the priming effects largely reflect lower-level form overlap between targets sharing a word pattern, and confirms that cross-modal priming reflects repeated access to morphemic representations shared by both prime and target.

Consistent with this, the priming effects in the [+Phonology] conditions in Experiments 1, 2, and 5 differ systematically from those observed in the [+WP] or [+Root] morphologically related conditions. Word pairs sharing two to three consonants that do not belong to the same underlying morpheme yield an average interference effect of about 20 ms across these three experiments. Although this interference reaches statistical significance only in Experiment 1, its recurrence across experiments suggests that lexical decisions to a target word are slower when the target follows a prime consisting of a similar but not identical root or word pattern.

Additional evidence comes from earlier studies of morphological processes in Arabic (e.g., Boudelaa & Marslen-Wilson, 2001, 2004a, 2005). All of these studies confirm in different ways that simple overlap in surface form between a prime and a target is not an effective means for generating facilitatory priming effects, of the sort typically observed for test pairs that share a common morpheme. One study (Boudelaa & Marslen-Wilson, 2004a) investigated the effects of allomorphic variation in root priming. These are experiments where the surface form of the root is altered, either by deletion or assimilation, as in cases like [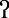
ittifaaqun]/[waafaqa] *agreement/agree*, where the underlying root {wfq} surfaces as [tfq] in the allomorphic form [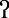
ittifaaqun]. These types of allomorphic variation do not affect priming, which is just as strong as priming between non-allomorphic prime-target pairs. What is important here is not whether prime and target are phonologically identical, but whether the phonological information present in the input points to the same underlying morpheme in both cases. A different study, using incremental masked priming (Boudelaa & Marslen-Wilson, 2005), demonstrates (in the orthographic domain) that the processing signatures of form overlap effects, in so far as they are detectable at all, are quite different from those involving morphological relations between prime and target – and similarly for purely semantic effects.

A further study, using a variety of priming techniques to probe the internal structure of the word pattern morpheme (Boudelaa & Marslen-Wilson, 2004a), suggests that morphological priming effects may be obtainable even when there is effectively *no* surface form overlap between prime and target. The Arabic verbal word pattern can potentially be broken down into two further morphemes – the vocalic melody (the sequence of vowels in the word pattern) and the CV-skeleton. The latter is a prosodic morpheme that specifies the phonological organisation of the surface form into a string of consonants (C) and vowels (V), and which carries important morpho-syntactic information (McCarthy, 1979, 1981).^17^

Primes and targets sharing a CV-skeleton, in tasks like masked priming, have essentially no orthographic material in common, since the two words have different roots (Arabic script is primarily consonantal) and will have different vowels, since they do not share a vocalic melody. Nonetheless, the pattern of priming for pairs sharing a CV-skeleton is comparable for masked priming and for tasks where the CV-skeleton is explicitly presented, as in cross-modal and auditory-auditory presentation. The CV-skeleton in masked priming is an abstract cognitive entity whose presence has to be inferred from a surface string made up primarily of consonants, and which then facilitates recognition of a target word where the same underlying morpheme has to be inferred from a surface string made up of a quite different string of consonants.

Semantic relationships are also an important potential source of priming in these experiments. Morphological relationships are established not only on the basis of shared form, but also on the basis of shared semantic content. Arabic surface forms sharing a word pattern also share many aspects of their grammatical properties, and words sharing a root usually relate more or less strongly to a common semantic field. There are several reasons, however, for rejecting an account of morphological priming in Arabic simply in terms of the semantic properties shared by prime and target.

The strongest arguments come from the finding of robust priming between prime and target pairs that share a common morpheme, but where one member of the pair is semantically opaque, and has no semantic relationship with the other. Where roots are concerned, there is consistent evidence that the strength of the priming effects between items sharing a root is not significantly dependent on semantic factors – in particular, on whether prime and target are in some measurable way semantically linked. For a pair like [mataa
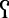
un]/[mut
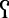
atun] *commodity/pleasure*, sharing the root {mt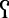}, the prime word [mataa
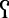
un] is semantically opaque, in the sense that the meaning of the whole form (*commodity*) is not synchronically consistent with the predominant meaning of the root (with the semantic field of *pleasure*). At the same time, the meaning of the prime word is not semantically related to the meaning of the target. The fact that robust priming is nonetheless obtained indicates (1) that the semantic opacity of the prime does not prevent it being decomposed to allow the extraction of the embedded root and (2) that despite the different semantic correlates of the root in prime and target, the same abstract linguistic entity is being accessed in the two cases. Similar results were observed in earlier research investigating a more abstract consonantal meaning unit, the bi-consonantal *etymon*, where priming was as strong for semantically transparent prime-target pairs as for pairs where one member was semantically opaque (Boudelaa & Marslen-Wilson, 2001).

Priming between words that are semantically but not morphologically related (e.g., [laððatun]/[mut
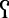
atun] *enjoyment/pleasure*) is generally much weaker than priming between items sharing a root. For Experiments 3 and 4, semantic priming averages 23 ms, which was not only not significant, but also much less than the 55 ms of facilitation seen for words sharing a root and a transparent semantic relationship (e.g., [mumti
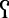
un]/[mut
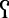
atun] *enjoyable/pleasure*).

A final piece of evidence that undermines a potential semantic account of morphemic priming results is the demonstration of strong priming by word patterns, which are bearers of morpho-syntactic and phonological information but not of semantic information *per se*. This is reflected in the semantic judgement pre-test described earlier, where subjects were instructed to rate semantic relationships among a set of 1200 words on a 1–9 scale, with 1 being unrelated and 9 highly related. Pairs of items sharing only a word pattern were rated as semantically very unrelated, averaging 1.40. Word patterns are fundamentally morphological entities subserving a productive linguistic process of word formation. There is no identifiable basis for ascribing priming between words sharing such units to any factors other than morphological. In these respects Arabic word patterns behave similarly to derivational affixes in English (e.g. *~ness* and *de~*), which generate significant priming between pairs such as *darkness/happiness* and *devalue/defrost* (Chateau, Knudsen, & Jared, 2002; Marslen-Wilson, Ford, Older, & Zhou, 1996; Post, Marslen-Wilson, Randall, & Tyler, 2008), but where the judged semantic relatedness between such forms remains very low.

In summary, and taking into account other comparisons using incremental masked priming (Boudelaa & Marslen-Wilson, 2005), this suggests parallel conclusions to those we reached above for possible phonological confounds. There is extensive evidence for Arabic that morphological priming effects cannot be attributed to confounds with semantic relatedness, that morphological priming is typically not modulated by the semantic relation between prime and target, and that semantic relatedness alone leads to weak and inconsistent priming effects. And if we do accept that the observed priming is indeed morphological in nature, then the behavioural evidence is consistently in favour of shared root and word-pattern morphemes, not imperfective stems.

## Conclusions and implications

The behavioural evidence for morphological effects in a language like Arabic is evidence for the role of genuinely morphological structure in the representation and processing of words in that language. An account of Arabic as a cognitive system needs to be able to represent underlying lexical elements as abstract morphemic units, where these representations abstract away both from the phonological features of their surface realisation as spoken forms and from the specific semantic properties of these forms, and where these representations are sufficiently linguistically abstract not to be bound to specific grammatical categories.

This conclusion, however, while placing firm constraints on the functional properties of a theory of the Arabic mental lexicon, does not in itself dictate the specific form that such an account should take – whether, for example, morphemes should be directly represented in a localist, possibly symbolically coded representational framework (e.g., Deutsch et al., 1998), or whether they should be theorised as nodes of regularity emerging as a result of learning in a more distributed cognitive system (e.g., Plaut & Gonnerman, 2000; Plaut, McClelland, Seidenberg, & Patterson, 1996; Rueckl, Mikolinski, Raveh, Miner, & Mars, 1997; Seidenberg 1987; Seidenberg & Gonnerman, 2000).

This is for two reasons. The first is that current behavioural data for Hebrew “weak” roots – as discussed in Velan, Frost, Deutsch, and Plaut (2005) by the protagonists of the two leading localist and distributive accounts – seem problematic for both approaches. These authors agree that the pattern of root priming for weak roots, where only two consonants are typically present in the surface form, is inconsistent both with key representational assumptions of the localist approach and with the statistical mechanisms used by the distributive approach to capture root-level regularities. Research on Arabic “weak” roots (Boudelaa & Marslen-Wilson, 2004a), while not identical to the studies reported by Velan et al. (2005), suggests that similar problems may arise for Arabic.

The second reason for caution in committing to an account of how morphological structure is captured in a model of the Arabic mental lexicon is that we have relatively little information at present about the neural underpinnings of the Arabic system. Accumulating fMRI evidence for Hebrew (e.g., Bick, Goelman & Frost, 2011) supports a distinction between semantic and morphological aspects of lexical processing, but is only indirectly relevant for Arabic, for the reasons outlined earlier. Neuropsychological and neuro-imaging research in English provides persuasive evidence for a dual neurobiological system underlying language function in general, distinguishing a fronto-temporal bilateral system, which supports general perceptual and interpretative processes underpinning speech comprehension, and a left hemisphere fronto-temporal system, selectively tuned to the processing of combinatorial grammatical sequences (Bozic, Tyler, Ives, Randall, & Marslen-Wilson, 2010; Marslen-Wilson & Tyler, 2007).

These two systems, furthermore, seem to interact differently with different morphological categories in English. Regular inflectional morphology selectively activates the left hemisphere system (e.g., Bozic et al., 2010), while derivationally complex words, even when highly transparent, seem to interact primarily with bilateral mechanisms for lexical access (Bozic & Marslen-Wilson, 2010; Bozic, Tyler, Su, Wingfield, & Marslen-Wilson, 2013). Given the very different character of Arabic morphemes – in particular the mixture of inflectional and derivational functions subserved by the word-pattern morpheme – we need to know how the processing functions associated with this morpheme relate to the two proposed underlying systems – and, indeed, how these two systems are realised more generally in the Arabic neurocognitive environment. A recent electrophysiological study, using electroencephalography (EEG), suggests that the processing of word pattern morphemes does show a left fronto-temporal distribution (Boudelaa et al., 2009), contrasting with a more fronto-central distribution for root morphemes. This is suggestive but inconclusive, given the restricted range of contrasts that could be explored in this experiment, and the coarse-grained localisation that is possible with EEG.

Finally, we should comment on the “universal” implications of the conclusions we have reached for Arabic, where we argue for a distinct representational and processing role for perceptually and linguistically abstract morphemic units. Clearly, this does not mean that every other language must therefore have similar properties in terms of the abstractness of underlying lexically related representations and how they combine to form surface words and phrases. What it does demonstrate is that it is possible for such forms of representation to emerge in an existing family of languages, which places the important constraint on psycholinguistic (and neurocognitive) accounts of human language that they are able to capture such an independent morphological dimension in their theoretical framework.

This in turn leads to the cross-linguistic question of why abstract morphological organisation may emerge more saliently in some languages and not in others. In the case of Arabic it is likely to reflect a complex synergy between the non-concatenative nature of Arabic word formation, where underlying morphemes never emerge as discrete surface phonological entities, and the importance of morphology in the language. In Arabic, morphological structure provides a domain of knowledge that is exceptionally consistent and regular both in terms of linguistic form and linguistic meaning (Boudelaa & Marslen-Wilson, 2005). The consistent recurrence in the language of roots and word patterns with similar meanings and similar grammatical implications means that they provide salient and relevant contingencies for the language learner to extract. As a result, the process of decomposing surface forms into roots and words patterns emerges naturally as an operation that helps the learner to make the right generalisations about the relevant units of the language, and where these generalisations are specified at the levels of abstraction induced by the non-concatenative properties of Arabic word formation.

These contingencies are clearly not going to be true of every human language – indeed, non-concatenative word formation may be unique to the Semitic language family. But if the morphological dimension is indeed a powerful and economical mechanism for organising the combination of lexical elements in a dynamic language system, then it seems likely that morphological structure, in forms appropriate to the language in question, will have emerged as a key feature of many, if not all, language systems worldwide.

## Notes

1. Here we adhere to the traditional notation of the word pattern morpheme which uses the letters “f”, “” and “l” as place holders for the first, second, and third letters of the root.

2. The “intensive” meaning is perhaps easier to illustrate in cases where the root (e.g., {lm} *knowledge*) combines both with the pattern {faail} to form the *agentive noun* [aalim] that is *someone who knows*, and with the pattern {faiil} to form the intensive noun [aliim] meaning *someone who is very knowledgeable*.

3. These are (ordered 2–10) {fa ala}, {faaala}, {afala}, {tafaala}, {tafaaala}, {infaala}, {iftaala}, {ifalla}, and {istafala}. They are considered to be augmented because they contain more consonantal and/or vocalic material than the unaugmented minimal pattern.

4. The term “estimative” is used to refer to forms which have an evaluative component, making a value judgement of some sort – in the case of [kaððaba] belie, attributing falsity.

5. The additional possibility, of “mediated” semantic priming where, for example, primes and targets such as *bull* and *milk* are related by a connecting mediator *cow*, will not be explicitly investigated since there is little evidence that such priming can be reliably obtained in tasks like lexical decision (e.g., Balota & Lorch, 1986).

6. These share the nominal word pattern {TA- -I-} where the dashes indicate the slots to be filled by the root letters.

7. These were the patterns {fuuulun}, {fiaalatun}, {fauulun}, {faiilun}, {falatun}, {fa aalun}, and {fulatun}.

8. A semantic control condition is not required here since, as discussed earlier, word patterns perform grammatical functions (which may affect semantic interpretation) but do not directly carry semantic information (in contrast to roots).

9. Three subjects mentioned at the end of the test that some words could be read differently, but that they chose to read them the way they did because it “*felt like the default reading*”. One of the subjects in this group read the word سفرة as [sufrah] *dining table* not as [safrah]’ *trip* as we intended it.

10. Note that the sequence [-un] shared by prime and target is an inflectional ending corresponding to the indefinite article. This ending is not included in the computation of amount of form overlap on the grounds that it is a constant in all forms used.

11. Following the request from a reviewer we also re-ran all of the RT analyses using mixed-effects regression models implemented in R. These delivered results that did not materially diverge from the results obtained from the ANOVA-based min-*F*’s analyses, and are not further reported here.

12. In all the experiments described here the number of syllables and of letters is highly positively correlated [*r* = .7, *n* = 96, *p* < .000]. Accordingly when we fitted a model to partial out the effects of prime length, we only did so for length in syllables. This is in any case more closely related to the actual duration of the primes, which were always spoken.

13. The linguistic term “effective” describes an act done to a person, or a state produced in him regardless of whether that act or state are self-inflicted or caused by someone else (Wright, 1995, p. 38).

14. In a subsequent mixed-effects analysis run on these data, this contrast between the two +Root conditions was not significant (*F*(1,451) = 0.81, *P* = 0.335). The further comparison between +R −S and –R +S remained marginally significant *F*(1,453) = 2.51, *P* = 0.06).

15. See note 13 above.

16. As Velan and Frost (2011) have recently demonstrated, not only are a substantial proportion of Hebrew nouns morphologically simple, in the sense that they are not analysable into a root and a word pattern at all (and are apparently processed as whole forms in the same way as English words such as *house* or *dog*), but also this seems to apply to the many forms with unique “non-productive” roots, which also pattern with the simple forms rather than those derived from a productive root.

17. McCarthy’s analysis, while applying persuasively to Arabic verbal word patterns, has not been convincingly applied across the board to nominal word patterns, especially where a morphemic interpretation of the vocalic melody is concerned.

## Appendix

**Appendix 1. Test items used in Experiment 1. For every item the Arabic script, an International Phonetic Association (IPA) transcription, and an English gloss are given.**

**Appendix 1** (*continued*)

## Appendix

**Appendix 2. Test items used in Experiment 2. For every item the Arabic script, an IPA transcription, and an English gloss are given.**

**Appendix 2** (*continued*)

## Appendix

**Appendix 3. Test items used in Experiment 3. For every item the Arabic script, an IPA transcription, and an English gloss are given.**

**Appendix 3** (*continued*)

## Appendix

**Appendix 4. Test items used in Experiments 4. For every item the Arabic script, an IPA transcription, and an English gloss are given.**

**Appendix 4** (*continued*)

## Appendix

**Appendix 5. Test items used in Experiment 5. For every item the Arabic script, an IPA transcription, and an English gloss are given.**

**Appendix 5** (*continued*)

## References

[cit0001] Allen M., Badecker W. (1999). Stem homograph inhibition and stem allomorphy: Representing and processing inflected forms in a multilevel lexical system. *Journal of Memory and Language*.

[cit0086] Baayen, R. H., Milin, P., Filipovic Durdevic, D., Hendrix, P., & Marelli, M. (2011).

[cit0002] Badawi M. S. (1973). *Levels of contemporary Arabic in Egypt*.

[cit0003] Balota D. A., Lorch R. F. (1986). Depth of automatic spreading activation: Mediated priming effects in pronunciation but not in lexical decision. *Journal of Experimental Psychology: Learning, Memory, and Cognition*.

[cit0004] Bat-El O., Shimron J. (2003). Semitic verb structure within a universal perspective. *Language processing and language acquisition in a root-based morphology*.

[cit0005] Benmamoun E. (1999). Arabic morphology: The central role of the imperfective. *Lingua*.

[cit0006] Benmamoun E., Shimron J. (2003). The role of the imperfective template in Arabic morphology. *Language processing and language acquisition in a root-based morphology*.

[cit0007] Berent I., Vaknin V., Marcus G. F. (2007). Roots, stems, and the universality of lexical representations: Evidence from Hebrew. *Cognition*.

[cit0087] Bick, A.S., Goleman, G., & Frost, R. (2011).

[cit0009] Bohas G., Guillaume J.-P. (1984). *Étude des Théories des Grammairiens Arabes* [A study of the Arab grammarian theories].

[cit0010] Boudelaa S., Saiegh-Haddad R. M. Joshi (2014). Is the Arabic mental lexicon morpheme-based or stem-based? Implications for spoken and written word recognition. *Handbook of Arabic literacy*.

[cit0011] Boudelaa S., Gaskell M. G. (2002). A re-examination of the default system for Arabic plurals. *Language and Cognitive Processes*.

[cit0012] Boudelaa S., Marslen-Wilson W. D. (2001). *The time-course of morphological, phonological and semantic processes in reading Modern Standard Arabic*.

[cit0013] Boudelaa S., Marslen-Wilson W. D. (2004a). Allomorphic variation in Arabic: Implications for lexical processing and representation. *Brain and Language,*.

[cit0088] Boudelaa, S., & Marslen-Wilson, W. D. (2004b).

[cit0014] Boudelaa S., Marslen-Wilson W. D. (2005). Discontinuous morphology in time: Incremental masked priming in Arabic. *Language and Cognitive Processes,*.

[cit0089] Boudelaa, S. & Marslen-Wilson, W. D. (2010).

[cit0015] Boudelaa S., Marslen-Wilson W. D. (2011). Productivity and priming: Morphemic decomposition in Arabic.. *Language and Cognitive Processes*.

[cit0016] Boudelaa S., Marslen-Wilson W. D. (2013). Morphological structure in the Arabic mental lexicon: Parallels between standard and dialectal Arabic. *Language and Cognitive Processes*.

[cit0017] Boudelaa S., Pulvermüller F., Hauk O., Shtyrov Y., Marslen-Wilson W. D. (2010). Arabic morphology in the neural language system: A mismatch negativity study. *Journal of Cognitive Neuroscience*.

[cit0018] Bozic M., Marslen-Wilson W. D. (2010). Neurocognitive contexts for morphological complexity: Dissociating inflection and derivation.. *Language and Linguistics Compass*.

[cit0019] Bozic M., Tyler L. K., Ives D. T., Randall B., Marslen-Wilson W. D. (2010). Bihemispheric foundations for human speech comprehension. *PNAS*.

[cit0020] Bozic M., Tyler L. K., Su L., Wingfield C., Marslen-Wilson W. D. (2013). Neurobiological systems for lexical representation and analysis in English. *Journal of Cognitive Neuroscience*.

[cit0021] Butterworth B., Butterworth B. (1983). Lexical representation.. *Language production*.

[cit0022] Cantineau J. (1950). *Racines et Schémes, Mélanges William Marçais* [Roots and word patterns.

[cit0023] Caramazza A., Laudanna A., Romani C. (1988). Lexical access and inflectional morphology. *Cognition,*.

[cit0024] Chateau D., Knudsen E. V., Jared D. (2002). Masked priming of prefixes and the influence of spelling–meaning consistency. *Brain and Language*.

[cit0025] Clahsen H., Fleischhauer E. (2014). Morphological priming in child German. *Journal of Child Language*.

[cit0026] Cohen, D. (1961).

[cit0027] Cohen, M. (1951).

[cit0029] Deutsch A., Frost R., Forster K. I. (1998). Verbs and nouns are organized and accessed differently in the mental lexicon: Evidence from Hebrew. *Journal of Experimental Psychology: Learning Memory, *&* Cognition*.

[cit0030] Deutsch A., Frost R., Pollatsek A., Rayner K. (2000). Early morphological effects in word recognition in Hebrew: Evidence from parafoveal preview benefit. *Language and Cognitive Processes*.

[cit0031] El-Dahdah A., Matar E. (1990). *A dictionary of universal Arabic grammar*.

[cit0032] Feldman L. B., Bentin S. (1994). Morphological analysis of disrupted morphemes: Evidence from Hebrew. *The Quarterly Journal of Experimental Psychology Section A*.

[cit0033] Feldman L. B., Frost R., Pnini T. (1995). Decomposing words into their constituent morphemes: Evidence from English and Hebrew. *Journal of Experimental Psychology: Learning, Memory, and Cognition*.

[cit0034] Frost R. (2011). Towards a universal model of reading. *The Behavioral and Brain Sciences*.

[cit0035] Frost R., Deutsch A., Gilboa O.,, Tannenbaum M., Marslen-Wilson W. (2000). Morphological priming: Dissociation of phonological, semantic, and morphological factors. *Memory *&* Cognition*.

[cit0036] Frost R., Forster K. I., Deutsch A. (1997). What can we learn from the morphology of Hebrew? A masked priming investigation of morphological representation. *Journal of Experimental Psychology: Learning, Memory, and Cognition*.

[cit0090] Frost, R., Kugler, T., & Forster, K. I. (2005).

[cit0091] Hammond, M. (1988).

[cit0092] Heath, J. (1987).

[cit0093] Heath, J. (2003).

[cit0039] Hilaal Y. (1990). Deriving from roots and word patterns. *Linguistica Communicatio*.

[cit0040] Hoberman R. D. (1988). Local and long-distance spreading in Semitic morphology. *Natural Language *&* Linguistic Theory,*.

[cit0041] Holes C. (1995). *Modern Arabic*.

[cit0042] Holyk G. G., Pexman P. M. (2004). The elusive nature of early phonological priming effects: Are there individual differences?. *Brain and Language*.

[cit0043] Idrissi A., Prunet J.-F., Béland R. (2008). On the mental representation of Arabic roots. *Linguistic Inquiry*.

[cit0044] Järvikivi J., Pyykkönen P., Niemi J. (2009). Exploiting degrees of inflectional ambiguity: Stem form and the time course of morphological processing. *Journal of Experimental Psychology: Learning, Memory and Cognition*.

[cit0045] Joanisse M. F., Seidenberg M. S. (1999). Impairments in verb morphology after brain injury: A connectionist model. *Proceedings of the National Academy of Science*.

[cit0047] Longtin C.-M., Segui J., Hallé P. A. (2003). Morphological priming without morphological relationship. *Language and Cognitive Processes*.

[cit0050] Marslen-Wilson W. D., Bozic M., Randall B. (2008). Early decomposition in visual word recognition: Dissociating morphology, form, and meaning. *Language and Cognitive Processes*.

[cit0051] Marslen-Wilson W. D., Ford M., Older L., Zhou X., Cottrell G. (1996). The combinatorial lexicon: Priming derivational affixes. *Proceedings of the 18th annual conference of the Cognitive Science Society*.

[cit0052] Marslen-Wilson W., Moss H. E., van Halen S. (1996). Perceptual distance and competition in lexical access. *Journal of Experimental Psychology: Human Perception *&* Performance*.

[cit0098] Marslen-Wilson, W. D., & Tyler, L. K. (2007).

[cit0053] Marslen-Wilson W., Tyler L. K., Waksler R., Older L. (1994). Morphology and meaning in the English mental lexicon. *Psychological Review*.

[cit0054] McCarthy J. J. (1979). *Formal problems in Semitic phonology and morphology*.

[cit0055] McCarthy J. J. (1981). A prosodic theory of non-concatenative morphology. *Linguistic Inquiry*.

[cit0056] McCarthy J. J., Prince A. S. (1990). Foot and word in prosodic morphology: The Arabic broken plural. *Natural Language and Linguistic Theory*.

[cit0058] Moss H. E., Marslen-Wilson W. D. (1993). Access to word meanings during spoken language comprehension: Effects of sentential semantic context. *Journal of Experimental Psychology: Learning, Memory, and Cognition*.

[cit0059] Orsolini M., Marslen-Wilson W. D. (1997). Universals in morphological representations: Evidence from Italian. *Language and Cognitive Processes*.

[cit0060] Post B., Marslen-Wilson W. D., Randall B., Tyler L. K. (2008). The processing of English regular inflections: Phonological cues to morphological structure. *Cognition*.

[cit0061] Plaut D. C., Gonnerman L. M. (2000). Are non-semantic morphological effects incompatible with a distributed connectionist approach to lexical processing?. *Language and Cognitive Processes*.

[cit0062] Plaut D. C., McClelland J. L., Seidenberg M. S., Patterson K. (1996). Understanding normal and impaired word reading: Computational principles in quasi-regular domains. *Psychological Review*.

[cit0099] R Development Core Team. (2014). http://www.R-project.org.

[cit0064] Rastle K., Brysbaert M. (2006). Masked phonological priming effects in English: Are they real? Do they matter?. *Cognitive Psychology*.

[cit0065] Rastle K., Davis M. H. (2008). Morphological decomposition based on the analysis of orthography. *Language and Cognitive Processes*.

[cit0066] Rastle K., Davis M. H., Marslen-Wilson W. D., Tyler L. K. (2000). Morphological and semantic effects in visual word recognition: A time-course study.. *Language and Cognitive Processes*.

[cit0067] Ratcliffe R. R. (1998). *The broken plural in Arabic and comparative Semitic: Allomorphy and analogy in non-concatenative morphology*.

[cit0095] Ratcliffe, R. R. (2004).

[cit0096] Ratcliffe, R. (2013).

[cit0068] Reid A. A., Marslen-Wilson W. D., Cutler A., McQueen J. M., Zondervan R. (2000). Complexity and alternation in the Polish mental lexicon.. *Proceedings of the workshop on spoken word access processes*.

[cit0069] Rueckl J. G., Mikolinski M., Raveh M., Miner C. S., Mars F. (1997). Morphological priming, fragment completion, and connectionist networks. *Journal of Memory and Language*.

[cit0071] Ryding K. C. (2005). *A reference grammar of Modern Standard Arabic*.

[cit0072] Schreuder R., Baayen R. H., Feldman L. B. (1995). Modelling morphological processing. *Morphological aspects of language processing*.

[cit0073] Seidenberg M., Coltheart M. (1987). Sublexical structure in visual word recognition: Access units or orthographic redundancy?. *Attention and performance XII*.

[cit0074] Seidenberg M. S., Gonnerman L. M. (2000). Explaining derivational morphology as the convergence of codes. *Trends in Cognitive Sciences*.

[cit0075] Shelton J. R., Martin R. C. (1992). How semantic is automatic semantic priming?. *Journal of Experimental Psychology: Learning, Memory, and Cognition*.

[cit0076] Shallice T., Cooper R. P. (2011). *The organisation of mind*.

[cit0077] Smolka E., Komlósi S., Rösler F. (2009). When semantics means less than morphology: The processing of German prefixed verbs. *Language and Cognitive Processes*.

[cit0078] Sonnenstuhl I.,, Eisenbeiss S., Clahsen H. (1999). Morphological priming in the German mental lexicon. *Cognition*.

[cit0080] Taft M., Forster K. I. (1975). Lexical storage and retrieval of prefixed words.. *Journal of Verbal Learning and Verbal Behavior*.

[cit0100] Vaknin, V., & Shimron, J. (2011).

[cit0081] Velan H., Frost R. (2011). Words with and without internal structure: What determines the nature of orthographic and morphological processing?. *Cognition*.

[cit0082] Velan H., Frost R., Deutsch A., Plaut D. C. (2005). The processing of root morphemes in Hebrew: Contrasting localist and distributed accounts. *Language and Cognitive Processes*.

[cit0083] Versteegh K. (1997). *The Arabic language*.

[cit0084] Wright W. (1995). *A grammar of the Arabic language*.

[cit0101] Zhou, X., & Marslen-Wilson, W. D. (1994).

